# Complete mitochondrial genomes of *Taenia multiceps*, *T. hydatigena *and *T. pisiformis*: additional molecular markers for a tapeworm genus of human and animal health significance

**DOI:** 10.1186/1471-2164-11-447

**Published:** 2010-07-22

**Authors:** Wan-Zhong Jia, Hong-Bin Yan, Ai-Jiang Guo, Xing-Quan Zhu, Yu-Chao Wang, Wan-Gui Shi, Hao-Tai Chen, Fang Zhan, Shao-Hua Zhang, Bao-Quan Fu, D Timothy J Littlewood, Xue-Peng Cai

**Affiliations:** 1 Key Laboratory of Veterinary Parasitology of Gansu Province, Key Laboratory of Zoonoses of Ministry of Agriculture, State Key Laboratory of Veterinary Etiological Biology, Lanzhou Veterinary Research Institute, CAAS, Lanzhou, Gansu Province 730046, The People's Republic of China; 2 Gansu Provincial Control for Animal Disease Control and Prevention, Lanzhou, Gansu Province 730046, The People's Republic of China; 3 Department of Zoology, The Natural History Museum, Cromwell Road, London SW7 5BD, UK

## Abstract

**Background:**

Mitochondrial genomes provide a rich source of molecular variation of proven and widespread utility in molecular ecology, population genetics and evolutionary biology. The tapeworm genus *Taenia *includes a diversity of tapeworm parasites of significant human and veterinary importance. Here we add complete sequences of the mt genomes of *T. multiceps*, *T. hydatigena *and *T. pisiformis*, to a data set of 4 published mtDNAs in the same genus. Seven complete mt genomes of *Taenia *species are used to compare and contrast variation within and between genomes in the genus, to estimate a phylogeny for the genus, and to develop novel molecular markers as part of an extended mitochondrial toolkit.

**Results:**

The complete circular mtDNAs of *T. multiceps*, *T. hydatigena *and *T. pisiformis *were 13,693, 13,492 and 13,387 bp in size respectively, comprising the usual complement of flatworm genes. Start and stop codons of protein coding genes included those found commonly amongst other platyhelminth mt genomes, but the much rarer initiation codon GTT was inferred for the gene *atp*6 in *T. pisiformis*. Phylogenetic analysis of mtDNAs offered novel estimates of the interrelationships of *Taenia*. Sliding window analyses showed *nad*6, *nad*5, *atp*6, *nad*3 and *nad*2 are amongst the most variable of genes per unit length, with the highest peaks in nucleotide diversity found in *nad*5. New primer pairs capable of amplifying fragments of variable DNA in *nad*1, *rrn*S and *nad*5 genes were designed *in silico *and tested as possible alternatives to existing mitochondrial markers for *Taenia*.

**Conclusions:**

With the availability of complete mtDNAs of 7 *Taenia *species, we have shown that analysis of amino acids provides a robust estimate of phylogeny for the genus that differs markedly from morphological estimates or those using partial genes; with implications for understanding the evolutionary radiation of important *Taenia*. Full alignment of the nucleotides of *Taenia *mtDNAs and sliding window analysis suggests numerous alternative gene regions are likely to capture greater nucleotide variation than those currently pursued as molecular markers. New PCR primers developed from a comparative mitogenomic analysis of *Taenia *species, extend the use of mitochondrial markers for molecular ecology, population genetics and diagnostics.

## Background

Tapeworms of the genus *Taenia *(Linnaeus, 1758) include parasites of mammals that use carnivores as definitive hosts, and herbivores (or omnivores) as intermediate hosts. Humans may be infected by both adult and larval forms (of various species), causing taeniosis or cysticercosis respectively. As with all species of tapeworm, transmission from host to host follows a trophic pathway via ingestion. However, species of *Taenia *are unique amongst the Cestoda in requiring two obligate mammalian hosts for transmission and life cycle completion; egg to herbivore, herbivore to carnivore, the cestode matures in the carnivore and releases fertilized eggs [1]. *Taenia *also enters humans trophically, through the inadvertent consumption of eggs or larval stages present in undercooked meat. Human *Taenia *species include *T. saginata *Goeze, 1782, *T. asiatica *Eom & Rim, 1993, and *T. solium *Linnaeus, 1758, with the following zoonotic species also found in humans: *T. taeniaeformis *(Batsch, 1786), *T. crassiceps *(Zeder, 1800), *T. multiceps *Leske, 1780, and *T. serialis *(Gervais, 1847). Species of *Taenia *cause significant health problems and considerable socio-economic losses when infecting humans and livestock [2]; human neurocysticercosis is the most common helminth infection of the central nervous system and has considerable societal impact in endemic areas [3-5]. The genus is widespread globally, with hotspots of prevalence in humans related to diet, social conditions, cultural practices and poverty. In the wild prevalence is dictated by specific predator-prey interactions, and there is little doubt their influence on host ecology is significant [6,7].

Historically, as an old genus, *Taenia *(Cestoda, Taeniidae) has become somewhat of a 'catch-all' taxon for tapeworm systematics, with upwards of 70 nominal species having been attributed to *Taenia*. Species circumscription based on morphology has been, and remains, problematic. Approximately 42 valid species and 3 subspecies are recognized currently [1], circumscribed predominantly on the basis of adult morphology; other names persist and largely arise from descriptions of larval stages or the use of some of the many synonyms [6,8-10]. There are conflicting estimates of phylogeny from morphology and various molecular markers, thus preventing a full understanding of the evolutionary history with their hosts through space and time. *Taenia *is one of only two genera in the Taeniidae. The other genus in the family, *Echinococcus*, is also of importance as it causes morbidity in humans and livestock [11-13]. As a result of their importance, species of these two genera have been studied extensively, but relatively little is understood about their biology in the context of molecular ecology, epidemiology or control. Much of this comes from an inability to quickly, or accurately, diagnose species or to track populations. In many endemic areas diseases caused by human *Taenia *are often categorized as 'neglected tropical diseases' [5].

As is widely recognized for many parasites, knowledge of life cycles, mechanisms and dynamics of transmission and infection, all form the basis for effective control strategies [12,14-16]. Molecular tools are increasingly used to develop these areas of knowledge, and here we take a comparative mitogenomic approach to evaluate mitochondrial (mt) DNAs as a source of new molecular markers.

Mitochondrial genes are amongst the most popular markers for molecular-based approaches to ecology, population genetics and evolutionary biology and have been popular targets for molecular-based methods of species identification [17-19]. Multiplex PCR approaches to diagnose mixed infections of *Taenia *are also being developed and are targeted for PCR-RFLP analysis using mt cytochrome c oxidase subunit I (*cox*1) and *cyt*b [20,21]. Certain genes and gene regions have become popular choices as representative mitochondrial markers, because they are bordered by regions of sequence conservation (e.g. *cox*1) and 'universal' (or at least broadly conserved) PCR primer sets can be readily designed. This is also the case for helminths, where mt gene fragments have been used routinely for population genetics, ecology and diagnostics [22,23]. More recently, the ability to readily characterize complete helminth mtDNAs, by means of long PCR and a variety of sequencing techniques [24,25], has prompted an assessment of entire genomes as a source for phylogenetic analysis [26-29], and their comparison to reveal variation within and between genes in order to develop novel (or optimise existing) molecular markers [30,31]. Mitochondrial genomes of bilaterian animals are short, circular DNA molecules typically 14-16 kb in length, without introns and with short intergenic regions. Gene content is highly conserved with typically 12 protein coding genes in Platyhelminthes (they lack the gene for ATP synthase subunit 8, *atp*8), two ribosomal subunits and 22 tRNAs. Mitochondrial gene rearrangement is not uncommon in flatworms, occurring within the genus *Schistosoma *[32,33], in some monogeneans [27] and appears to be different in at least some turbellarians [34]. Evidence suggests that gene order is otherwise generally conserved in tapeworms (Cestoda) and flukes (Trematoda) [35].

Complete mtDNAs offer variation over multiple levels of organization, from gene content and gene order to variation in amino acids and nucleotides, offering opportunities to resolve both recent and ancient divergence events. Here we characterize the complete mt genomes of *T. multiceps *Leske, 1780, *T. hydatigena *Pallas, 1766 and *T. pisiformis *(Bloch, 1780), each of intrinsic interest. Coenurosis is a debilitating disease caused by the metacestode (larval forms) of *T. multiceps *and is common in sheep and other herbivores. Humans may also be infected with the metacestode occasionally [36]; infections occur when eggs are ingested via the fecal-oral route of transmission. Infective eggs hatch, and the liberated oncospheres cross the membrane of the small intestine and migrate within the body, typically ending up in the central nervous system, mesentery and visceral organs [37,38]. Within the host's tissues, the oncospheres mature into coenuri or cysticerci causing the metacestodiasis. Amongst carnivores, each of these 3 species is cosmopolitan in canines and *T. hydatigena *and *T. pisiformis *are also found in felines. In China, sheep are the most common hosts for metacestodes of *T. multiceps *and *T. hydatigena*, and lagomorphs are the most common hosts of *T. pisiformis *tapeworms; their distribution across China and surrounding countries is extensive [39-41]. Each of the species characterized here is found globally, and mixed infections with other taeniids are common. For accurate diagnosis, there is a need to identify individual species from mixed infections of tapeworms whether in intermediate or definitive hosts. Differentiating adult worms using morphology alone requires taxonomic expertise, but to differentiate amongst mixed populations of eggs and larvae requires molecular techniques [42]. Once developed, molecular techniques can be readily applied to portions of adult worms, larvae, eggs and environmental samples and should aid in accurate, rapid identification.

Among the genus *Taenia*, complete mt genome sequences are already available for *T. asiatica*, *T. saginata*, *T. solium *and *T. crassiceps *[32,43,44]. We use these sequences with the new data to achieve three goals. Firstly, we evaluate the potential for complete mt genomes in estimating the phylogeny of the genus and revealing its evolutionary history. *Taenia *is a species-rich genus with a widespread distribution but there are conflicting estimates of phylogeny from morphology and various molecular markers. Secondly, we use comparative mitogenomics to highlight regions of nucleotide variation amongst *Taenia *species to investigate whether mitochondrial gene fragments currently used as molecular markers offer the best regions for characterization, whether for species recognition or other molecular-based applications. Third, we take an *in silico *approach to developing PCR primer pairs designed to amplify short fragments of mtDNAs for all species (for which entire mt genomes have been characterized), with a view to providing primers that will work for all *Taenia *species capturing high levels of variation in mtDNAs, and we test some of these primers to demonstrate their efficacy.

## Results and Discussion

### *General features of the mt genome of 3 *Taenia *species*

The mtDNAs of cestodes are similar to those of other eumetazoans with respect to length, gene composition, and the structures of their tRNA and rRNA molecules [32,43,44]. The complete mtDNA sequences of *T. multiceps*, *T. hydatigena *and *T. pisiformis *were 13,693 bp, 13,492 bp and 13,387 bp in length, respectively, of which *T. pisiformis *mtDNA sequence is the smallest among the Platyhelminthes studied to date (Table 1). Yap et al. [45] reported that restriction mapping of the *T. hydatigena *mt genome suggested it to be 17.6 kb, but no published complete mt genomes sequences from taeniids have been more than 15 kb in length. The size of *T. pisiformis *mtDNA in this study is also among the smallest reported for metazoans; only 5 other metazoan mtDNAs are known to be smaller [35]. In general, gene size, organization, codon use and nucleotide content are very similar amongst the newly sequenced *Taenia *species and those already characterized.

**Table 1:** Positions and gene lengths in the mitochondrial genomes of *T. multiceps *(Tm)*, T. hydatigena *(Th) and *T. pisiformis *(Tp)

Genes	Positions and lengths of nucleotide sequences (bp)			Initiation and termination codons			Anticodons
	Tm	Th	Tp	Tm	Th	Tp	Tm/Th/Tp
*trn*G	1-65 (65)	1-66 (66)	1-72 (72)				TCC
*cox*3	69-713 (645)	70-714 (645)	76-719 (643)	ATG/TAA	GTG/TAA	GTG/T	
*trn*H	721-794 (74)	717-783 (67)	720-790 (71)				GTG
*Cyt*b	798-1865 (1068)	787-1854 (1068)	794-1861(1068)	ATG/TAG	ATG/TAA	ATG/TAA	
*nad*4L	1882-2142 (261)	1864-2124 (261)	1866-2126 (261)	ATG/TAA	ATG/TAG	ATG/TAG	
*nad*4	2109-3362 (1254)	2091-3344 (1254)	2093-3346 (1254)	ATG/TAA	ATG/TAA	ATG/TAA	
*trn*Q	3372-3436 (65)	3352-3411 (60)	3347-3409 (63)				TTG
*trn*F	3436-3499 (64)	3409-3472 (64)	3409-3471 (63)				GAA
*trn*M	3496-3560 (65)	3469-3532 (64)	3468-353 1(64)				CAT
*atp*6	3568-4083 (516)	3540-4055 (516)	3538-4050 (516)	ATG/TAG	ATG/TAA	GTT/TAA	
*nad*2	4086-4967 (882)	4059-4937 (879)	4053-4928 (876)	GTG/TAA	ATG/TAA	ATG/TAA	
*trn*V	4976-5041 (66)	4953-5014 (62)	4933-4994 (62)				TAC
*trn*A	5064-5127 (64)	5033-5096 (64)	5008-5070 (63)				TGC
*trn*D	5134-5200 (67)	5102-5168 (67)	5081-5143 (63)				GTC
*nad*1	5205-6098 (894)	5174-6067 (894)	5148-6044 (897)	ATG/TAA	ATG/TAA	ATG/TAG	
*trn*N	6102-6169 (68)	6071-6137 (67)	6053-6119 (67)				GTT
*trn*P	6188-6252 (65)	6156-6218 (63)	6135-6197 (63)				TGG
*trn*I	6254-6320 (67)	6220-6284 (65)	6198-6260 (63)				GAT
*trn*K	6332-6396 (65)	6286-6350 (65)	6267-6330 (64)				CTT
*nad*3	6398-6745 (348)	6351-6698 (348)	6331-6677 (347)	ATG/TAA	GTG/TAA	ATG/T(A)	
*trn*S(AGN)	6752-6810 (59)	6699-6757 (59)	6678-6737 (60)				GCT
*trn*W	6820-6887 (68)	6765-6827 (63)	6739-6802 (64)				TCA
*cox*1	6891-8513 (1623)	6831-8450 (1620)	6806-8425 (1620)	ATG/TAA	ATG/TAA	ATG/TAA	
*trn*T	8499-8561 (63)	8436-8498 (63)	8411-8473 (63)				TGT
*rrn*L	8562-9539 (978)	8499-9472 (974)	8474-9443 (970)				
*trn*C	9540-9600 (61)	9473-9531 (59)	9444-9501(58)				GCA
*rrn*S	9601-10340 (740)	9532-10258 (727)	9502-10229 (728)				
*cox*2	10341-10919 (579)	10259-10840 (582)	10230-10814 (585)	ATG/TAA	ATG/TAG	ATG/TAG	
*trn*E	10935-11002 (68)	10847-10914 (68)	10820-10885 (66)				TTC
*nad*6	11003-11455 (453)	10917-11369 (453)	10887-11339 (453)	GTG/TAG	ATG/TAG	ATG/TAA	
*trn*Y	11468-11530 (63)	11377-11441 (65)	11345-11406 (62)				GTA
SNR	11531-11603 (73)	11442-11505 (64)	11407-11473 (67)				
*trn*L(CUN)	11604-11671 (68)	11506-11571 (66)	11474-11542 (69)				TAG
*trn*S(UCN)	11720-11777 (58)	11605-11662 (58)	11574-11634 (61)				TGA
*trn*L(UUN)	11792-11856 (65)	11664-11727 (64)	11637-11698 (62)				TAA
*trn*R	11887-11944 (58)	11732-11788 (57)	11700-11754 (55)				ACG
*nad*5	11946-13517 (1572)	11789-13357 (1569)	11758-13323 (1566)	ATG/TAA	ATG/TAA	ATG/TAA	
LNR	13518-13693 (176)	13358-13492 (135)	13324-13387 (64)				

Start and stop codons for protein-coding genes as well as lengths of their predicted amino acid sequences and tRNA gene anticodons (starting from *trn*G). SNR and LNR refer to short and long non-coding regions respectively.

Each of the 3 mtDNAs contained 36 genes lacking ATP synthase subunit 8 gene (*atp*8), of which 12 were protein-coding genes (*atp*6, cytochrome c oxidase subunits 1, 2 and 3 [*cox*1*-cox*3], cytochrome b [*cyt*b] and nicotinamide dehydrogenase subunits 1-6 [*nad*1*-nad*6] and 4L [*nad*4L]), 22 were tRNA genes (two coding for leucine, and two coding for serine) and the small [*rrn*S] and large [*rrn*L] subunits of rRNA, as found in other Platyhelminthes [46]. The mt gene arrangements of these 3 cestodes are identical to that of other taeniids and only slightly different from that of *Hymenolepis diminuta *by the reversal in order of *trn*S2 and *trn*L1 [32,47].

All genes are transcribed in the same direction. Some genes overlap in the mtDNAs, which is also true for other flatworm species. The 3'-end of *nad*4L overlaps with the 5'-end of *nad*4 as observed in all flatworms; length of overlaps is the same (34 bp) in all *Taenia *species [48,49]. However, in the published annotations of *E. multilocularis *(NC_000928) and *T. solium *(NC_004022), *nad*4L and *nad*4 do not overlap, which needs to be revised according to our alignment; in these cases *nad4*L ends on legitimate stop codons before *nad*4 begins [43,50] and there are no indications from our alignment that they are incorrect. Additional gene overlaps were observed between 3'-end of *cox*1 and the 5'-end of *trn*T (15 bp), between *trn*Q and *trn*F, and *trn*F and *trn*M (1-5 bp). A full list of inferred gene boundaries and lengths are given in Table 1.

As with other flatworm mtDNAs, the nucleotide compositions of the new sequences are biased toward T and A, with T being the most favored nucleotide and C the least favored [46]. AT-richness in *T. multiceps *is 71.3% (24.4% A, 46.9% T, 20.3% G and 8.4% C), in *T. hydatigena *is 70.9% (24.9% A, 46.0% T, 20.3% G and 8.8% C) - the lowest for any taeniid studied to date - and in *T. pisiformis *73.2% (27.7% A, 45.5% T, 18.0% G and 8.8% C), shown in Additional file 1. AT-bias in *Taenia *species is higher than amongst *Echinococcus *species, but not unusual amongst cestodes [49]. Amongst protein coding genes AT-content varies from 68.2 to 74.7%. Amongst *Taenia *species cytochrome *c *oxidase genes tend to have lower, or the lowest, AT-content [32,43] and long (LNR) and short non-coding regions (SNR) tend to have the highest AT-content (73.5-82.1%).

Flatworms employ an unusual mitochondrial code for translating codons into amino acids [51,52]. GTG is being proposed as an alternative initiation codon to ATG in *nad*3 and *cox*3 genes of *T. hydatigena*, in *cox*3 of *T. pisiformis *and in *nad*2 and *nad*6 genes of *T. multiceps*. We also infer an even more unusual start codon, GTT, in *atp*6 of *T. pisiformis*; this has been suggested for *cox*1 of *E. granulosus *(G4 strain, AF346403) [15] and *H. diminuta *(NC_002767) [47]. To avoid potential sequencing errors, we confirmed GTT as a start codon in *T. pisiformis *by sequencing the region (double-stranded) five times using PCR products directly and cloned recombinant plasmids from two isolates of *T. pisiformis *cysticerci. Two abbreviated stop codons were also found in mt protein-coding genes *nad*3 and *cox*3 of *T. pisiformis*, as found in *nad*3 and *cox*3 of *Diphyllobothrium latum*, *D. nihonkaiense *[49,53,54] and *Spirometra erinaceieuropaei *(GenBank NC_011037), shown in Additional file 2.

### Structure of tRNAs

Using ARWEN [55] nearly all 22 tRNAs, with the conventional secondary structures, were found for each mt genome. Remaining tRNAs were identified using alignment and inferred secondary structures (Additional file 3). Mitochondrial tRNA genes were 55-74 bp long, and the predicted secondary structures of 18 of these had typical clover-leaf shapes with paired dihydrouridine (DHU) arms. The *trn*C, *trn*R and two *trn*S tRNAs contained a predicted secondary structure with the TΨC arm and loop but lacked the DHU arm and loop, as found in some other platyhelminth mtDNAs [15,54]. Anticodons in all 22 tRNAs for all *Taenia *species are conserved without mutations. The first two nucleotides preceding the anticodons are almost always thymidine, the first nucleotide following the anticodons is usually adenosine or guanosine, and the second is guanosine.

### Structure of SNR and LNR

The non-coding regions of the mt genomes of *T. multiceps*, *T. hydatigena *and *T. pisiformis *comprised 2 major regions: a short non-coding region (SNR) or NR1 (73, 64 and 67 nucleotides, respectively) and a long non-coding region (LNR) or NR2 (176, 135 and 64 nucleotides, respectively). Among published taeniid mtDNAs the LNR of *T. pisiformis *is the shortest. SNR and LNR were located between *trn*Y and *trn*L1 (UCN), and *nad*5 and *trn*G, respectively.

In the LNR regions of *T. multiceps *and *T. hydatigena *mt genomes and the NR2 of *T. pisiformis *mt genome, there were sets of short inverted repeats (Additional file 4). One could be folded into a hairpin structure characterized by 4 to 6 consecutive G_C or inconsecutive base pairs at the upside of the stem. Although the SNR and NR1 were short, there was at least one inverted sequence in these regions. A significantly stable potential secondary structure containing a stem-loop could be folded using SNR or NR1. Similar inverted sequences and stem-loop secondary or hairpin structure are also found in these two regions of other taeniid mt genomes [43,50]. Besides LNR of *T. multiceps *mt genome, one inverted repeat sequence (5'-ATATATATACGGGGG-CCCCGTATATATAT-3') was displayed in LNR of *T. asiatica*, *T. saginata*, *T. solium *and *T. crassiceps *mt genomes, and another similar inverted sequence (5'-ATATATAGAGAGAGGGG-CCCCTCTCTCTATATAT-3') appeared also in NR2 of *T. pisiformis *mtDNA. Stable hairpin structures, such as those identified in the LNR and the SNR of taeniid mtDNAs [43,47], likely contain the origins of replication and transcription [56].

### *Phylogeny of *Taenia

Using a variety of shared characteristics including those defining adult morphology (particularly the organization of male and female reproductive organs) and larval structure, Hoberg et al. [6] compiled 27 characters for 30 species of *Taenia*. Parsimony analysis found 4 equally parsimonious trees, and the consensus tree has since been used to infer patterns of intermediate and definitive host association [1]. Inferred switching between carnivore definitive hosts led Hoberg [1] to conclude that ecological shifts have been greatly responsible for the patterns of radiation in *Taenia*. This study highlighted the likely role of climatological and ecological perturbation in influencing host-parasite associations in the genus. The morphology-based phylogeny has not been supported by partial molecular data sets from subsets of taxa, but establishing a reliable phylogeny for the genus clearly has important consequences.

Early attempts to infer molecular phylogenies of *Taenia *have been hampered by the availability of different species, and the application of relatively crude models of molecular evolution. Nevertheless, two studies provide perspective in assessing estimates from new complete mtDNA and the morphological data. De Queiroz and Alkire [57] compiled available partial *cox*1 data and partial nuclear large subunit (28S) rDNA data to infer a phylogeny including 11 species of *Taenia*. Lavikainen et al. [58] sampled multiple isolates of 9 *Taenia *species for partial *cox*1 and partial *nad*1 genes; this built on data collected by Gasser et al. [59]. In each of these studies, all the species for which complete mtDNAs are now available were included, thus allowing direct comparison of phylogenetic estimates of 7 species of *Taenia*.

**Figure 1:**
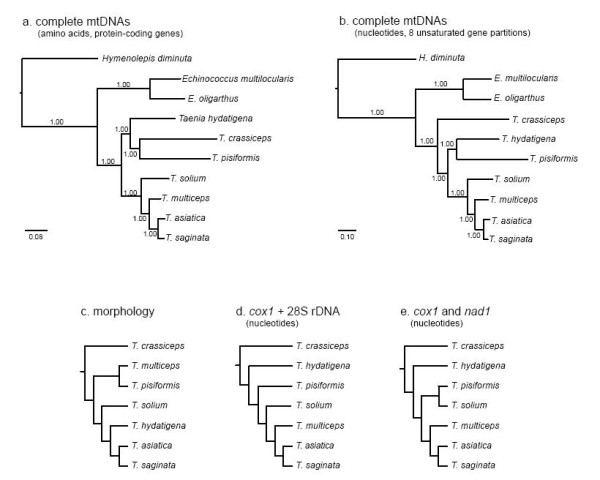
**Phylogenetic estimates of the interrelationships of 7 *Taenia *species for which complete mtDNAs are available, based on: a. Bayesian analysis of all alignable amino acids of protein coding genes from complete mtDNAs, b. Bayesian analysis of all alignable, unsaturated nucleotides from complete mtDNAs (8 partitions; see text), c. cladistic analysis of morphology **[6]**, d. maximum likelihood and parsimony analysis of partial *cox*1 and partial nuclear 28S rDNA **[57]**, and e. maximum likelihood analysis of partial *cox*1 and partial *nad*1 **[58]. Scale bars in a. and b. indicate number of substitutions per site; c, d and e are drawn as cladograms with equal branch lengths to indicate topologies only as they are adapted from larger analyses.

Alignment of all protein-coding genes of *Taenia *mtDNAs and outgroups provided 3,324 unambiguously aligned amino acids available for phylogenetic analysis, of which 1679 were variable and 1,009 parsimony-informative. Alignment of all nucleotides provided 11,393 unambiguously aligned sites, of which 9,378 were variable and 5,901 parsimony-informative. To avoid potential problems of saturation, only coding positions that were shown to be unsaturated were included in the nucleotide analysis; leaving 7,655 unambiguously aligned sites of which 3,680 were variable and 2,365 parsimony-informative. Bayesian analysis yielded phylogenies with maximal nodal support throughout the amino acid (Figure 1a), and nucleotide (Figure 1b) estimates. The interrelationships of *Taenia *based on complete mtDNAs were different between estimates from amino acids and nucleotides, and previously estimated phylogenies based on either morphology (Figure 1c) or molecular data (Figure 1d and 1e). Consistent among all phylogenetic estimates is the sister group status between *T. asiatica *and *T. saginata*, as expected, since these species are known to be very closely related [44]; recent evidence has demonstrated on-going hybridization between these species [60]. Molecular estimates from complete mtDNAs, partial mt and nuclear gene fragments consistently reveal *T. multiceps *as most closely related to *T. saginata *+ *T. asiatica*. The placement of *T. multiceps *in this clade suggests a common intermediate host of members of the Suidae (pig family). Additional taxon sampling is required to further test the inferences of intermediate and definitive host switching in the genus made by Hoberg [1]. Beyond this clade, no two molecular estimates are identical with respect to the remaining species. The interrelationships of *T. hydatigena*, *T. crassiceps *and *T. pisiformis *in the mtDNA analysis are different from one another, but together they clearly form a clade (united by each using members of the Canidae as definitive hosts), in contrast to previous estimates. It is noteworthy that the nodal support in previous studies, using only small fragments of DNA, was generally very poor throughout the estimated phylogenies (not shown), in contrast to the very robust support offered by complete mtDNA amino acid analysis. Although additional evidence is required to resolve the interrelationships of the *T. crassiceps*, *T. hydatigena *and *T. pisiformis *clade, the analysis of amino acids suggests additional mtDNAs of *Taenia *species can be incorporated readily to provide a wider estimate of taeniid phylogeny.

Further studies are required to elucidate the source(s) of incongruence between the amino acid and nucleotide estimates, but it may be the result of added homoplasy from the ribosomal genes, tRNA genes and non-coding regions included in the analysis. Whether the more sophisticated Bayesian model (mtZoa) used for the amino acid analysis has provided a more accurate estimate of interrelationships of *Taenia*, which requires further testing, additional sampling of nuclear genes, such as complete 18S and complete 28S rDNA [61], may be useful in providing 3 independent estimates (morphology, mitochondrial and nuclear DNA) of phylogeny. Parsimony mapping of morphological characters detailed by Hoberg et al. [6] onto the mtDNA amino acid phylogeny, using only the 7 *Taenia *species common to both studies, suggests 3 morphological characters might support nodes in the novel topology in Figure 1a; these included characters 11 (localization of the metacestode in the intermediate host), 19 (route of the vas deferens) and 20 (number of layers in the testes). However, a full assessment of the morphological data set, by means of an independent molecular-based phylogeny is premature without additional sampling of *Taenia *species.

### *Nucleotide variation within and between *Taenia *mtDNAs*

**Figure 2:**
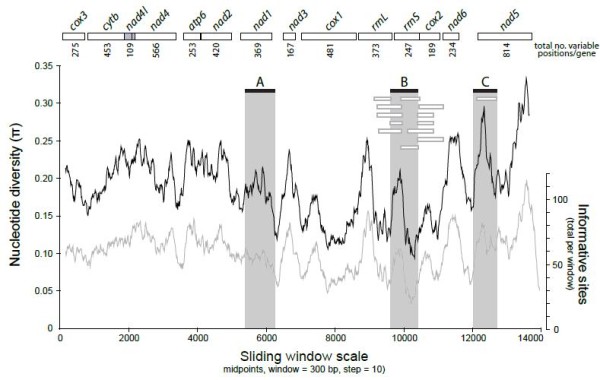
**Sliding window analyses of the alignment of complete mtDNAs of 7 species of *Taenia***. The black line shows the value of nucleotide diversity (π) in a sliding window analysis of window size 300 bp with step size 10, the value is inserted at its mid-point. The grey line shows the total number of phylogenetically informative sites (under the principles of parsimony) in the alignment for any given 300 bp window, also plotted at a step size of 10. Gene boundaries are indicated with an indication of the total number of variable positions per gene; *nad4*L and *nad*4 are overlapping. Solid black boxes indicate the fragments amplified by 3 primer pairs (A, B and C) designed from this alignment using PriFi [67]; Alternative primers are indicated by open grey boxes.

Sliding window analysis of the nucleotide alignment of all available *Taenia *mtDNAs provided an indication of nucleotide diversity (π) within and between mitochondrial genes (Figure 2). The plot readily shows the high degree of nucleotide variation within and between genes amongst the aligned *Taenia *genomes for any given window of 300 bp (π ranges from 0.094 to 0.333), and the possibility that hitherto untested regions of mtDNAs may be of utility. GenBank holds relatively few mitochondrial markers for a few species of *Taenia*, used variously for population genetics and diagnostics, but include partial fragments of *cox*1, *cyt*b [60,62], *nad*1 [63], *rrn*S and *atp*6 (unpublished data available on GenBank). Sliding window analysis (and computation of number of variable positions per unit length of gene), indicates that genes with high sequence variability included *nad*6 (0.517), *nad*5 (0.517), *atp*6 (0.490), *nad*3 (0.480) and *nad*2 (0.479). Genes with the lowest sequence variability (per unit length) include *cox*1 (0.296), *cox*2 (0.323) and *rrn*S (0.330). Sequence variability within genes appears to be highest, with notable peaks and troughs of π, in *cox*1, *rrn*S and *nad*5. A plot of the number of phylogenetically informative sites in the same alignment, and using the same sliding window parameters, revealed a very close correlation between sequence variability and phylogenetically informative sites (Figure 2), allowing π to be used as a readily available proxy for phylogenetic signal; DnaSP [64] calculates π easily, but the plot of phylogenetically informative positions is more tedious. Nucleotide variability within species tends to reflect the same pattern observed amongst closely related species (e.g. within a genus; Littlewood, unpublished). Combined, these results suggest alternative gene regions are worthy of consideration in developing new genetic markers for phylogenetics, population genetics and diagnostics. Current mt genes used as targets for multiplexed PCR-RFLP based approaches to diagnostics [20,21,65] include *cox*1 and *cyt*b; *cox*1 is also a chosen target for a novel loop-mediated isothermal amplification method of diagnosis [66]. Although relatively easy to amplify routinely, *cox*1 is amongst the slowest evolving, and least variable of genes available in the entire mtDNA suggesting that more reliable, or at least more informative, markers should be considered for future work, especially diagnostics involving mixed infections or other *Taenia *species.

### In silico *prediction of novel mitochondrial markers for *Taenia *species*

**Figure 3:**
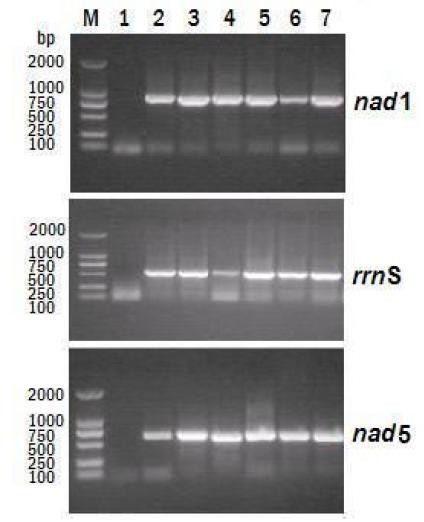
**Results of testing new PCR primers against various taeniid templates**. PCR amplified fragments are examined in 1% agarose gels stained with Ethidium bromide: Lane M, DL2000 molecular marker; Lane 1, no template DNA; Lane 2 to 7, *T. multiceps*, *T. hydatigena*, *T. pisiformis*, *T. asiatica*, *T. saginata *and *T. solium*.

PriFi [67] was used to predict primer pairs capable of amplifying 500-800 bp lengths of any *Taenia *mtDNA. This length was chosen since amplicons of ~650 bp are amenable to a diversity of qualitative and quantitative analyses (e.g. restriction digests, sequencing). As with many algorithms developed for primer design, changing search and filtering parameters can yield primers of variable quality. Figure 2 illustrates the positions of all primers found with one particular setting, chosen to minimize nucleotide degeneracy whilst maximizing inclusiveness across *Taenia *species. Three primer pairs were selected for their high score (meeting or exceeding most of the primer selection criteria), and were found in *nad*1, *rrn*S and *nad*5, genes of medium to high nucleotide diversity, but likely exceeding the variability offered by popular gene regions such as *cox*1 and *cyt*b. Primer pairs were: A (*nad*1) forward 5'-CARTTTCGTAAGGGBCCWAAWAAGGT, reverse 5'-CCAATTTCYTGAAGTTAACAGCATCA; B (*rrn*S) forward 5'- AGGGGATAGGRCACAGTGCCAGCATCTGCGG, reverse 5'- AATTCATTTAAAGTTACCTTGTTACGACTTACCTC; C (*nad*5) forward 5'- TATATGAGTTAGTTTTAAGCATTAATTATGG, reverse 5'- GGAAAHCTAGCACTCTTDGTAA. Primers A, B and C yield fragments of *Taenia *mitochondrial DNA approximately 871, 558 and 670 bp respectively and capture 241, 146 and 326 variable positions in the total alignment of the *Taenia *mtDNAs; equivalent to 14.1% of the overall variable nucleotide positions (n = 5,770). Efficacy of the 3 primer pairs herein designed was tested with PCR method. As expected, the 3 target PCR amplification bands were observed for the genus *Taenia *(Figure 3).

## Conclusions

*Taenia *species are important socio-economic parasites because they have a significant impact on human, domestic and wild animal health. Complete mtDNAs of *Taenia *provide an example of the extremely economic and compact organization of flatworm mtDNAs. Analysis of amino acids of mtDNAs provides a well-supported phylogeny for *Taenia *species, at odds with estimates from morphology; *T. multiceps *is more closely related to *T. saginata *and *T. asiatica *than to *T. solium*, and that *T. hydatigena*, *T. crassiceps *and *T. pisiformis *form a monophyletic group. Additional mitochondrial markers for the study of *Taenia *highlighted by this study include those for which high nucleotide variability has been identified (*nad*6, *nad*5, *atp*6, *nad*3 and *nad*2). *In silico *analysis of all available mtDNAs of *Taenia*, suggested new primers capable of capturing higher levels of mitochondrial nucleotide diversity than those currently used for diagnosis or molecular ecology. These data have implications for molecular diagnostic methods, epidemiological investigations and systematic studies of taeniid parasites.

## Methods

### Parasites and DNA extraction

Single tapeworms each of *T. multiceps *and *T. pisiformis *tapeworm were collected for DNA extraction and sequencing. *T. multiceps *was collected from a dog infected experimentally with *Coenurus cerebralis *from naturally infected sheep (Gansu Provincial Huangcheng Wool Sheep Breeding Farm). A single cysticercus of *T. pisiformis *was isolated from a naturally infected rabbit (at a slaughterhouse in Shandong Province) in our laboratory, and a cyst of the same species was collected from a rabbit in Henan Province. One *T. hydatigena *cyst was collected from the abdominal cavity of a sheep at a slaughterhouse in Qinghai Province. Other adult worms, *T. asiatica*, *T. saginata *and *T. solium *from patients were also used for genomic DNA extraction. Fragments from the tapeworms and a protoscolex from the cyst were washed with cold phosphate-buffered saline and frozen in liquid nitrogen. Genomic DNA was isolated using Genomic DNA Purification Kit (Puregene^® ^DNA Purification System, Gentra Systems, Minneapolis, Minnesota, USA) according to the manufacturer's instructions.

### Amplification of mtDNA fragments

The total length of the mt genome was amplified in 9 overlapping fragments using EX TaqTM polymerases with 3'-5' exonuclease proofreading activity (Takara Biotechnology Co. Ltd, Dalian, China) using total genomic DNA purified from a single cyst or worm as the template. The overlapping fragments of *T. multiceps*, *T. hydatigena *and *T. pisiformis *mtDNAs were amplified using nine pairs of oligonucleotide primers (Additional file 5), designed according to the conserved regions from published complete mtDNA sequences of taeniid cestodes. All PCR reactions comprised ~20-40 ng of the genomic DNA in a 50 μl reaction containing 1.5 U *Taq *polymerase, 10 mM Tris-HCl pH9, 50 mM KCl, 2 mM MgCl_2_, 200 μM of each dNTP. PCR amplifications each proceeded with 35 cycles of 94°C for 1 min, 52°C for 45 s, 72°C for 2 to 4 min depending on product length. The amplicons were then cloned into the pGEM-T Easy vector (Promega Co., Winsconsin, USA). At least 3 clones from each amplicon were double-stranded sequenced.

### Sequencing and assembling of DNA fragments

All sequencing was performed using terminator-based cycle sequencing with BigDye chemistry (Applied Biosystems, Foster City, CA, USA) on an ABI 3730 or 373 DNA sequencer (Applied Biosystems) at Shanghai Sangon or Takara Biotechnology Co. Amplicons were sequenced to completion by primer walking. Chromatograms were visualized using reports were analyzed using Chromas 2.33 software http://www.technelysium.com.au, and sequences were assembled using CUGI's New CAP3 Server online (The Clemson University Genomics Institute, from http://www.genome.clemson.edu/) [68]. Sequence data were analyzed with the SeqMan and MegAlign programs, and the consensus sequence of each amplicon was used as the final sequence (DNASTAR Inc., Madison, WI, USA).

Nucleotide sequences identified in this study have been submitted to GenBank, and the accession numbers for *T. multiceps*, *T. hytigena *and *T. pisiformis *mtDNAs are GQ228818, GQ228819 and GU569096, respectively. The published mtDNA sequences for other Cestoda used in this study include: *T. solium *(NC_004022), *T. saginata *(NC_009938), *T. asiatica *(NC_004826), *T. crassiceps *(NC_002547), *Echinococcus multilocularis *(NC_000928), *E. oligarthrus *(NC_000928) and *Hymenolepis diminuta *(NC_002767).

### Prediction of protein-coding genes

The protein-coding regions were identified using BLAST searches, ORF finder of DNAStar and comparisons with other sequences of Platyhelminthes available in the GenBank database http://www.ncbi.nlm.nih.gov/BLAST/. Genetic codes were based on translation table nine and those in cestodes [49,52].

### *Prediction of tRNAs and genes for *rrn*L and *rrn*S*

Putative tRNA genes were identified using the software ARWEN http://130.235.46.10/ARWEN/[55], combined with visual inspection of aligned mtDNAs and tRNA genes. Genes for *rrn*L and *rrn*S were identified from sequence similarities to the published cestode mitochondrial rRNA genes [43]. Putative stem-loop structures of non-coding mitochondrial regions (LNR and SNR) were inferred using the program RNAstructure v. 4.6) [69,70].

### Mitochondrial gene arrangement

Mitochondrial gene arrangements were compared by eye for gene adjacencies in all pairwise combinations for *T. multiceps*, *T. hydatigena *and *T. pisiformis *according to *T. solium*, *T. saginata*, *T. asiatica*, *T. crassiceps *and *E. multilocularis*.

### Alignment and phylogenetic analysis

Two other taeniids, early divergent members of *Echinococcus*, *E. multilocularis *and *E. oligarthus *[48], and *Hymenolepis diminuta *were selected as suitable outgroups. Nucleotides of all *Taenia *mtDNAs and outgroups were aligned initially by eye, in frame where appropriate for protein-coding genes, using MacClade [71]. Amino acids translations were inferred using the flatworm mitochondrial code (Table nine, GenBank http://www.ncbi.nlm.nih.gov/Taxonomy/Utils/wprintgc.cgi?mode=c#SG9) [52], exported and handled in a separate MacClade file. Nucleotide and amino acid alignments consisted of 11,983 and 3404 positions respectively, and each were exported to the Gblocks server http://molevol.cmima.csic.es/castresana/Gblocks_server.html) running Gblocks 0.91 b [72] under default parameters, to remove poorly aligned positions. Gblocks removed 4.9% (n = 590 nucleotides) and 2.3% (n = 80 amino acids) of the nucleotide and amino acid alignments respectively. For the nucleotide data set, individual genes were first scrutinized to see whether they were overly saturated. Using Xia et al.'s test [73], as implemented in Dambe[74], individual genes were tested using all sites, and for each protein-coding gene 1^st^, 2^nd ^and 3^rd ^positions were checked individually. The following 8 gene partitions passed the test and were included in the phylogenetic analysis (^1,2 ^indicates sites 1 + 2 included only, otherwise all sites included): *cox*3, *cyt*b, *nad*4^1,2^, *nad*2, *nad*1^1,2^, *cox*1, *cox*2^1,2 ^and *nad*5. Both ribosomal RNA genes, *atp*6, *nad*3, *nad*4L and *nad*6 were significantly saturated and were excluded. The 8 unstaurated gene partitions provided a total of 7,655 nucleotides available for phylogenetic analysis.

Bayesian analyses of the nucleotide and amino acid analyses were each carried out using MrBayes, v.3.1.2 [75]. Following the recommendations of Rota-Stabelli et al. [76], for the amino acid data set the mtZoa model was applied (settings were rates = gamma, ngammacat = 5, aamodel = fixed (rtrev) ); two chains (temp = 0.2) were run for 5,000,000 generations and sampled every 1,000 generations. Using the unsaturated gene partitions only, Modeltest 3.7maxX [77] was used to estimate a suitable model for nucleotide substitution; this was equivalent to GTR+I+G and settings were nst = 6, rates = invgamma, ngammacat = 4. Four chains (temp = 0.2) were run for 5,000,000 generations and sampled every 1,000 generations. For each analysis convergence was assessed using Tracer v 1.4 [78], with a discarded burn-in period of 5,000 trees. Posterior probabilities provided evidence of nodal support. All trees were rooted against *Hymenolepis diminuta.*

The morphological data set of Hoberg et al. [6] was incorporated into a MacClade [71] file for each of the 7 *Taenia *species studied here, to enable character mapping onto alternative tree topologies offered by alternative data sets. Only unambiguous changes were traced to determine whether novel tree topologies from molecular data were supported by morphological characters.

### Sliding window analysis of nucleotide variation

The complete alignment of nucleotides of the 7 *Taenia *mtDNAs was used to effect sliding window analyses using DnaSP v.5 [64]. A sliding window of 300 bp and steps of 10 bp was used to estimate nucleotide diversity (π) for the entire alignment. Nucleotide diversity for the entire alignments was plotted against midpoint positions of each window, and gene boundaries indicated. PAUP* v. 4.0b10 [79] was used to determine the position of phylogenetically informative positions under the principle of parsimony, and the sum of these sites was calculated using a sliding window approach with the same parameters (window size = 300 bp; step size = 10 bp) and plotted on the same graph.

### *Design of novel mitochondrial markers for *Taenia *and their applications in PCR*

Using the same complete nucleotide alignment of mtDNAs, but with outgroups and deletions common to all taxa removed, novel PCR primers were sought using PriFi [67]. This software looks for primer pairs that fit given criteria, with the added benefit of being designed on the basis of all taxa in the alignment. PriFi only accepts relatively short alignments and so 4,000 bp segments of the complete alignment were subjected to analysis; each segment overlapped the next by 1,000 bp and a segment including both ends of the linear alignment (to complete the mtDNA circle) was also analyzed. Settings differed from default parameters as follows: minimum melting temperature = 45.0˙C, critical melting temperature = 55.0˙C, minimum number of 3'-end matches = 3, optimal primer length interval = (22, 35 bp), optimal PCR product length interval = (400, 500, 800, 1,000 bp), minimum product length = 350 bp, conservation window length = 50 bp.

All PCR reactions comprised ~20-40 ng of the genomic DNA in a 50 μl reaction containing 1.25 U *Taq *polymerase, 10 mM Tris-HCl pH9, 50 mM KCl, 2 mM MgCl_2_, 200 μM of each dNTP, and 0.2 μM each primer. PCR amplifications each proceeded with 35 cycles of 94°C for 1 min 30 s, 52~57°C for 30 s, 72°C for 1 min. Primer pairs were: A for *nad*1, B for *rrn*S and C for *nad*5.

## Authors' contributions

WZJ and HBY equally conceived and designed the research plan, and performed the majority of the study and analyzed the data, and contributed to drafting of the manuscript. AJG, YCW, FZ and SHZ performed part of the study, and provided technical assistance. WGS and FBQ conducted the related animal infection experiments. XQZ and HTC contributed to partial analysis of the data and the revision of the manuscript. DTJL took the lead on analysis of the data and drafting of the manuscript. XPC participated in all aspects of the study, supervised the research and provided funds. All authors read and approved the final manuscript.

## Supplementary Material

Additional file 1 **Comparison of A+T content (%) of the protein-coding, tRNA, rRNA genes of mitochondrial genomes of *Taenia *species studied to date**. Click here for file

Additional file 2 **Properties of protein-coding genes, lengths of NR1 and NR2 regions and AT content of cestode mtDNAs**. Click here for file

Additional file 3 **Predicted secondary structures of tRNAs from A: *T. multiceps*, B: *T. hydatigena *and C: *T. pisiformis *mtDNAs**. Click here for file

Additional file 4 **Sequences and putative structures of LNR and SNR found in the *T. multiceps*, *T. hydatigena *and *T. pisiformis *mtDNAs**. Arrows show inverted repeats, which is similar to that of *E. multilocularis *[50]. Nucleotides in the box are shared by two inverted repeats. A: LNR and SNR sequences of *T. multiceps *mtDNA and their predicted secondary structures. B: LNR and SNR sequences of *T. hydatigena *mtDNA and their predicated secondary structures. C: NR1 and NR2 sequences of *T. pisiformis *mtDNA and their predicated secondary structures. Click here for file

Additional file 5 **Primers for amplification of mtDNA fragments and their position in the mt genome of *T. pisiformis *(Tp)**. Click here for file
