# Supplementary material for: Complete mitochondrial genomes of Taenia multiceps, T. hydatigena and T. pisiformis: additional molecular markers for a tapeworm genus of human and animal health significance
Source: BMC Genomics. 2010 Jul 22;11:447. doi: 10.1186/1471-2164-11-447 (PMC3091644; doi:10.1186/1471-2164-11-447)
Supplement: Additional file 1 — Comparison of A+T content (%) of the protein-coding, tRNA, rRNA genes of mitochondrial genomes of Taenia species studied to date. [file 1471-2164-11-447-S1.DOC]

**Additional file 1. Comparison of A+T content (%) of the protein-coding, tRNA, rRNA genes of mitochondrial genomes of *Taenia* species studied to date.**

| **Regions** | ***T. multiceps*** | | | | | ***T. hydatigena*** | | | | | ***T. pisiformis*** | | | | | ***T. crassiceps*** | | | | |
| --- | --- | --- | --- | --- | --- | --- | --- | --- | --- | --- | --- | --- | --- | --- | --- | --- | --- | --- | --- | --- |
|  | T | A | G | C | AT | T | A | G | C | AT | T | A | G | C | AT | T | A | G | C | AT |
| **Protein-coding** | 48.8 | 22.6 | 20.6 | 8.0 | 71.4 | 48.1 | 22.9 | 20.6 | 8.4 | 71.0 | 47.4 | 26.0 | 18.1 | 8.5 | 73.4 | 50.5 | 23.5 | 18.9 | 7.1 | 74.0 |
| ***rrn*L** | 41.2 | 28.6 | 20.3 | 10.0 | 69.7 | 40.7 | 28.3 | 20.5 | 10.5 | 69.0 | 40.9 | 32.3 | 17.2 | 9.6 | 73.2 | 44.0 | 30.0 | 17.0 | 9.1 | 74.0 |
| ***rrn*S** | 42.7 | 29.2 | 18.9 | 9.2 | 71.9 | 39.8 | 29.9 | 20.2 | 10.2 | 69.6 | 40.5 | 32.8 | 17.5 | 9.2 | 73.4 | 44.0 | 30.9 | 16.6 | 8.5 | 74.9 |
| **tRNAs** | 40.4 | 29.5 | 20.0 | 10.1 | 69.9 | 39.6 | 31.9 | 18.9 | 9.6 | 71.5 | 38.9 | 31.3 | 19.0 | 10.7 | 70.2 | 42.6 | 30.2 | 17.6 | 9.5 | 72.8 |
| **SNR** | 45.2 | 32.9 | 11.0 | 11.0 | 78.1 | 43.8 | 35.9 | 9.4 | 10.9 | 79.7 | 37.3 | 44.8 | 9.0 | 9.0 | 82.1 | 38.5 | 41.5 | 7.7 | 12.3 | 80.0 |
| **LNR** | 38.6 | 40.3 | 11.9 | 9.1 | 79.0 | 35.6 | 35.6 | 17.0 | 11.9 | 71.1 | 37.5 | 37.5 | 12.5 | 12.5 | 75.0 | 38.7 | 40.7 | 10.3 | 10.3 | 79.4 |
| **Full genome** | 46.9 | 24.4 | 20.3 | 8.4 | 71.3 | 46.0 | 24.9 | 20.3 | 8.8 | 70.9 | 45.5 | 27.7 | 18.0 | 8.8 | 73.2 | 48.7 | 25.4 | 18.3 | 7.6 | 74.0 |
|  |  | | | | |  | | | | |  | | | | |  | | | | |
| **Regions** | ***T. asiatica*** | | | | | ***T. saginata*** | | | | | ***T. solium*** | | | | | **All the seven *Taenia* species** | | | | |
|  | T | A | G | C | AT | T | A | G | C | AT | T | A | G | C | AT | T | A | G | C | AT |
| **Protein-coding** | 49.2 | 22.4 | 21.0 | 7.5 | 71.5 | 47.3 | 24.2 | 20.6 | 7.9 | 71.5 | 48.5 | 23.6 | 20.0 | 7.8 | 72.1 | 48.8 | 23.3 | 20.0 | 7.8 | 72.2 |
| ***rrn*L** | 40.9 | 28.5 | 20.6 | 10.0 | 69.4 | 41.1 | 27.9 | 21.2 | 9.9 | 68.9 | 41.5 | 29.1 | 19.9 | 9.5 | 70.6 | 41.5 | 29.2 | 19.5 | 9.8 | 70.7 |
| ***rrn*S** | 41.3 | 29.1 | 20.0 | 9.6 | 70.5 | 42.1 | 29.0 | 20.0 | 9.0 | 71.0 | 42.4 | 30.1 | 18.7 | 8.8 | 72.5 | 41.8 | 30.1 | 18.8 | 9.2 | 72.0 |
| **tRNAs** | 41.2 | 29.7 | 19.7 | 9.5 | 70.9 | 40.9 | 29.5 | 19.7 | 9.9 | 70.5 | 41.1 | 29.9 | 19.2 | 9.9 | 70.9 | 40.7 | 30.3 | 19.2 | 9.9 | 71.0 |
| **SNR** | 41.4 | 37.1 | 8.6 | 12.9 | 78.6 | 40.9 | 37.9 | 10.6 | 10.6 | 78.8 | 39.7 | 33.8 | 14.7 | 11.8 | 73.5 | 41.0 | 37.6 | 10.2 | 11.2 | 78.7 |
| **LNR** | 36.9 | 42.1 | 13.1 | 8.0 | 79.0 | 36.5 | 40.3 | 13.2 | 10.1 | 76.7 | 35.4 | 40.1 | 12.5 | 12.0 | 75.5 | 37.0 | 39.9 | 12.8 | 10.3 | 76.9 |
| **Full genome** | 47.1 | 24.3 | 20.7 | 8.0 | 71.4 | 49.4 | 22.4 | 20.8 | 7.4 | 71.7 | 46.7 | 25.4 | 19.7 | 8.3 | 72.1 | 46.9 | 25.0 | 19.8 | 8.2 | 71.9 |
