# Supplementary material for: Complete mitochondrial genomes of Taenia multiceps, T. hydatigena and T. pisiformis: additional molecular markers for a tapeworm genus of human and animal health significance
Source: BMC Genomics. 2010 Jul 22;11:447. doi: 10.1186/1471-2164-11-447 (PMC3091644; doi:10.1186/1471-2164-11-447)
Supplement: Additional file 2 — Properties of protein-coding genes, lengths of NR1 and NR2 regions and AT content of cestode mtDNAs. [file 1471-2164-11-447-S2.DOC]

**Additional file 2.** Properties of protein-coding genes, lengths of NR1 and NR2 regions and AT content of cestode mtDNAs.

| Gene/  Regions | Species or Strains *** | | | | | | | | | | | | | | | | | | | | |
| --- | --- | --- | --- | --- | --- | --- | --- | --- | --- | --- | --- | --- | --- | --- | --- | --- | --- | --- | --- | --- | --- |
|  | **T.m** | **T.h** | **T.p** | **T.a** | **T.sa** | **T.so** | **T.c** | **EgG1** | **EgG4** | **EgG5** | **EgG6** | **EgG7** | **EgG8** | **E.m** | **E.o** | **E.s** | **E.v** | **H.d** | **D.l** | **D.n** | **S.e** |
| No. of amino acids | | | |  |  |  |  |  |  |  |  |  |  |  |  |  |  |  |  |  |  |
| *cox*3 | 214 | 214 | 214 | 214 | 214 | 214 | 214 | 215 | 215 | 215 | 215 | 215 | 215 | 215 | 215 | 215 | 215 | 216 | 214 | 214 | 214 |
| *cob* | 355 | 355 | 355 | 355 | 355 | 355 | 357 | 355 | 355 | 355 | 355 | 355 | 355 | 355 | 355 | 355 | 355 | 365 | 368 | 368 | 369 |
| *nad*4L | 86 | 86 | 86 | 86 | 86 | 86 | 86 | 86 | 86 | 86 | 86 | 86 | 86 | 86 | 86 | 86 | 86 | 86 | 86 | 86 | 86 |
| *nad*4 | 417 | 417 | 417 | 417 | 417 | 417 | 417 | 419 | 419 | 419 | 419 | 419 | 419 | 419 | 420 | 419 | 419 | 415 | 416 | 416 | 417 |
| *atp*6 | 171 | 171 | 170 | 171 | 171 | 171 | 170 | 170 | 170 | 170 | 170 | 170 | 170 | 171 | 170 | 170 | 170 | 171 | 169 | 169 | 171 |
| *nad*2 | 293 | 292 | 291 | 293 | 293 | 293 | 292 | 293 | 293 | 293 | 293 | 293 | 293 | 293 | 294 | 293 | 293 | 293 | 292 | 292 | 290 |
| *nad*1 | 297 | 297 | 298 | 297 | 297 | 297 | 297 | 297 | 297 | 297 | 297 | 297 | 297 | 297 | 297 | 298 | 297 | 296 | 296 | 296 | 296 |
| *nad*3 | 115 | 115 | 115 | 115 | 115 | 115 | 115 | 115 | 115 | 115 | 115 | 115 | 115 | 115 | 115 | 115 | 115 | 115 | 115 | 115 | 115 |
| *cox*1 | 540 | 539 | 539 | 539 | 539 | 539 | 537 | 535 | 534 | 535 | 535 | 535 | 535 | 535 | 535 | 535 | 532 | 518 | 521 | 521 | 521 |
| *cox*2 | 192 | 193 | 194 | 191 | 194 | 193 | 194 | 193 | 193 | 191 | 191 | 191 | 191 | 193 | 193 | 193 | 193 | 192 | 189 | 189 | 189 |
| *nad*6 | 150 | 150 | 150 | 150 | 150 | 150 | 150 | 151 | 151 | 151 | 151 | 151 | 151 | 151 | 151 | 151 | 151 | 152 | 152 | 152 | 155 |
| *nad*5 | 523 | 522 | 521 | 522 | 522 | 522 | 522 | 523 | 523 | 523 | 523 | 523 | 523 | 524 | 523 | 524 | 523 | 524 | 522 | 522 | 522 |
|  |  |  |  |  |  |  |  |  |  |  |  |  |  |  |  |  |  |  |  |  |  |
| Inferred initiation codon | | | |  |  |  |  |  |  |  |  |  |  |  |  |  |  |  |  |  |  |
| *cox*3 | ATG | GTG | GTG | ATG | ATG | ATG | GTG | ATG | ATG | ATG | ATG | ATG | ATG | ATG | ATG | ATG | ATG | ATG | GTG | GTG | GTG |
| *cob* | ATG | ATG | ATG | ATG | ATG | ATG | ATG | ATG | ATG | ATG | ATG | ATG | ATG | ATG | ATG | ATG | ATG | ATG | ATG | ATG | ATG |
| *nad*4L | ATG | ATG | ATG | ATG | ATG | ATG | ATG | GTG | GTG | ATG | ATG | ATG | GTG | GTG | ATG | ATG | GTG | ATG | ATG | ATG | ATG |
| *nad*4 | ATG | ATG | ATG | ATG | GTG | ATG | ATG | ATG | ATG | ATG | GTG | GTG | GTG | ATG | ATG | GTG | ATG | ATG | ATG | ATG | ATG |
| *atp*6 | ATG | ATG | *GTT* | ATG | GTG | ATG | GTG | ATG | ATG | ATG | ATG | ATG | ATG | ATG | ATG | GTG | ATG | ATG | ATG | ATG | ATG |
| *nad*2 | GTG | ATG | ATG | ATG | ATG | ATG | ATG | ATG | ATG | ATG | ATG | ATG | ATG | ATG | GTG | ATG | ATG | ATG | ATG | ATG | ATG |
| *nad*1 | ATG | ATG | ATG | ATG | ATG | ATG | ATG | GTG | ATG | ATG | ATG | ATG | ATG | ATG | ATG | GTG | ATG | ATG | ATG | ATG | ATG |
| *nad*3 | ATG | GTG | ATG | ATG | ATG | ATG | GTG | ATG | ATG | ATG | ATG | ATG | ATG | ATG | ATG | ATG | ATG | ATG | ATG | ATG | ATG |
| *cox*1 | ATG | ATG | ATG | ATG | ATG | ATG | ATG | ATG | GTG | GTG | ATG | ATG | ATG | ATG | ATG | ATG | ATG | *GTT* | ATG | ATG | ATG |
| *cox*2 | ATG | ATG | ATG | ATG | ATG | ATG | ATG | GTG | GTG | GTG | GTG | GTG | GTG | GTG | GTG | ATG | GTG | ATG | ATG | ATG | ATG |
| *nad*6 | GTG | ATG | ATG | ATG | ATG | ATG | GTG | ATG | ATG | ATG | ATG | ATG | ATG | ATG | ATG | ATG | ATG | ATG | ATG | ATG | ATG |
| *nad*5 | ATG | ATG | ATG | ATG | ATG | ATG | ATG | ATG | ATG | ATG | ATG | ATG | ATG | ATG | ATG | ATG | ATG | ATG | ATG | ATG | ATG |
|  |  |  |  |  |  |  |  |  |  |  |  |  |  |  |  |  |  |  |  |  |  |
| Inferred ternination codon | | | | |  |  |  |  |  |  |  |  |  |  |  |  |  |  |  |  |  |
| *cox*3 | TAG | TAA | T | TAG | TAG | TAG | TAG | TAG | TAG | TAA | TAA | TAA | TAA | TGA | TAG | TAG | TAG | TAG | T | T | T |
| *cob* | TAG | TAA | TAA | TAA | TAA | TAA | TAA | TAA | TAA | TAG | TAG | TAG | TAG | TAA | TAA | TAG | TAA | TAG | TAA | TAA | TAA |
| *nad*4L | TAA | TAG | TAG | TAG | TAG | TAA | TAG | TAA | TAA | TAA | TAA | TAA | TAA | TAG | TAA | TAG | TAG | TAG | TAA | TAA | TAG |
| *nad*4 | TAA | TAA | TAA | TAG | TAG | TAG | TAG | TAG | TAA | TAG | TAG | TAG | TAG | TAG | TAA | TAA | TAA | TAG | TAG | TAG | TAG |
| *atp*6 | TAG | TAA | TAA | TAA | TAA | TAA | TAA | TAG | TAG | TAG | TAG | TAG | TAG | TAG | TAG | TAG | TAG | TAG | TAG | TAG | TAA |
| *nad*2 | TAA | TAA | TAA | TAA | TAA | TAA | TAG | TAG | TAG | TAG | TAG | TAG | TAG | TAG | TAA | TAG | TAG | TAG | TAG | TAG | TAG |
| *nad*1 | TAA | TAA | TAG | T | T | T | TAG | TAA | TAA | TAA | TAA | TAA | TAA | TAG | TAG | TAA | TAA | TAG | T(A) | T(A) | TAA |
| *nad*3 | TAA | TAA | T(A) | TAG | TAA | TAG | TAG | TAG | TAA | TAA | TAA | TAA | TAA | TAA | TAA | TAG | TAA | TAG | T | T | T |
| *cox*1 | TAA | TAA | TAA | TAA | TAA | TAG | TAG | TAG | TAG | TAG | TAG | TAG | TAG | TAG | TAG | TAG | T | T | TAG | TAG | TAG |
| *cox*2 | TAA | TAG | TAG | TAG | TAG | TAG | TAG | TAG | TAG | TAG | TAG | TAG | TAG | TAG | TAG | TAG | TAA | TAA | TAA | TAA | TAA |
| *nad*6 | TAG | TAG | TAA | TAA | TAA | TAG | TAA | TAG | TAG | TAG | TAG | TAG | TAG | TAA | TAG | TAG | TAG | TAA | TAG | TAG | TAA |
| *nad*5 | TAA | TAA | TAA | TAA | TAA | TAA | TAA | TAA | TAA | TAA | TAA | TAA | TAA | TAA | TAA | TAA | TAA | TAG | TAA | TAA | TAA |
|  |  |  |  |  |  |  |  |  |  |  |  |  |  |  |  |  |  |  |  |  |  |
| Length of overlap between *nad*4L and *nad*4 (bp) | | | | | | |  |  |  |  |  |  |  |  |  |  |  |  |  |  |  |
|  | 34 | 34 | 34 | 34 | 34 | 34 | 34 | 40 | 40 | 40 | 40 | 40 | 40 | 40 | 40 | 40 | 40 | 40 | 40 | 40 | 40 |
|  |  |  |  |  |  |  |  |  |  |  |  |  |  |  |  |  |  |  |  |  |  |
| Lengths of non-coding regions (bp) | | | | | |  |  |  |  |  |  |  |  |  |  |  |  |  |  |  |  |
| NR1 | 73 | 64 | 67 | 70 | 66 | 68 | 65 | 66 | 66 | 183 | 183 | 183 | 183 | 183 | 185 | 182 | 183 | 183 | 222 | 224 | 204 |
| NR2 | 176 | 135 | 64 | 176 | 159 | 192 | 194 | 184 | 178 | 176 | 178 | 178 | 178 | 177 | 176 | 178 | 181 | 443 | 188 | 331 | 174 |
|  |  |  |  |  |  |  |  |  |  |  |  |  |  |  |  |  |  |  |  |  |  |
| AT content of complete mitochondrial genome (%) | | | | | |  |  |  |  |  |  |  |  |  |  |  |  |  |  |  |  |
|  | 71.3 | 70.9 | 73.2 | 71.4 | 71.7 | 72.1 | 74.0 | 67.1 | 67.9 | 67.8 | 67.6 | 67.7 | 67.6 | 69.0 | 69.3 | 68.4 | 67.2 | 71.0 | 68.3 | 67.8 | 66.4 |

Results based on GenBank accessions, publications and alignment analyses.

* Abrreviations: T.m: *T*. *multiceps*; T.h: *T*. *hydatigena*; T.p: *T*. *pisiformis*; T.so: *T*. *solium*; T.sa: *T*. *saginata*, T.as: *T*. *asiatica*; T.c: *T*. *crassiceps*; EgG1: *E. granulosus* G1 type; EgG4-EgG8: *E. granulosus* G4-G8; E.m: *E. multilocularis*; E.o: *E. oligarthrus*; E.s: *E. shiquicu*s; E.v: *E. vogeli*; H.d: *Hymenolepis diminuta*; D.l: *Diphyllobothrium latum*; D.n: *D. nihonkaiense*; S.e: *Spirometra erinaceieuropaei*.
