# Supplementary figures and images for: Complete mitochondrial genomes of Taenia multiceps, T. hydatigena and T. pisiformis: additional molecular markers for a tapeworm genus of human and animal health significance
Source: BMC Genomics. 2010 Jul 22;11:447. doi: 10.1186/1471-2164-11-447 (PMC3091644; doi:10.1186/1471-2164-11-447)

Additional file 3

A *T. multiceps*


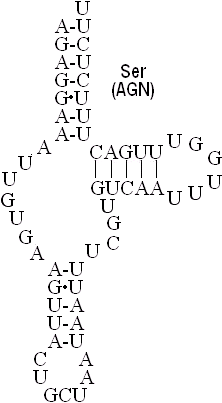

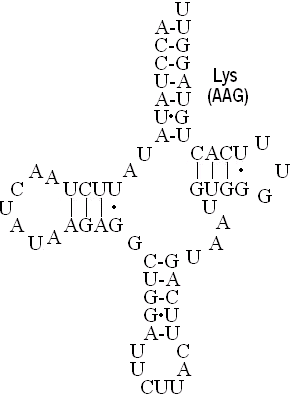

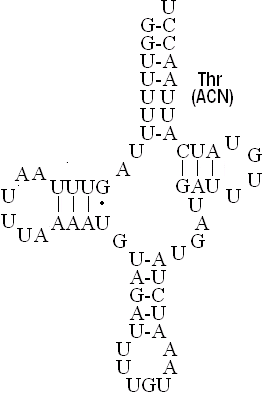

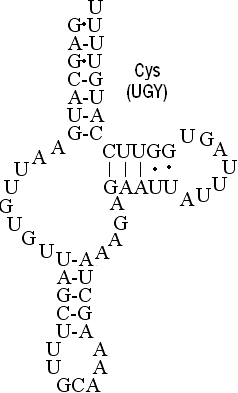

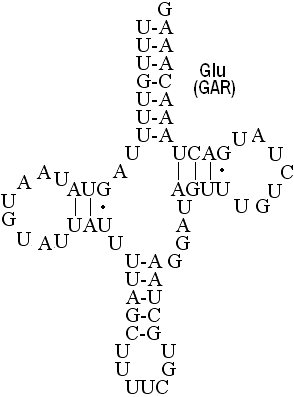

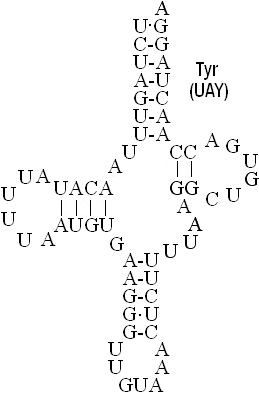

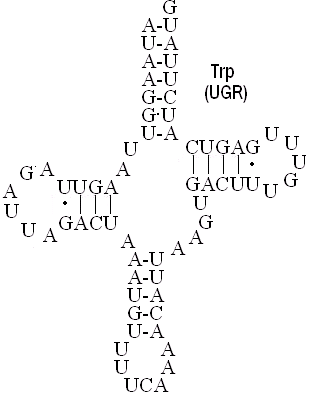

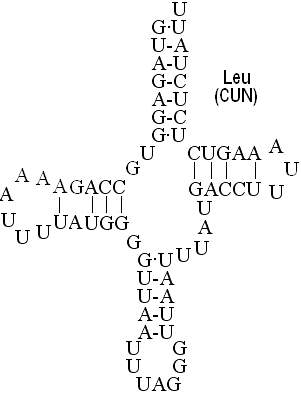

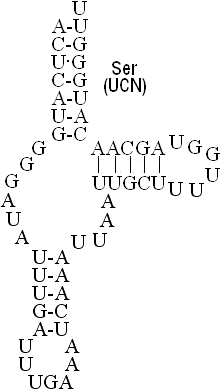

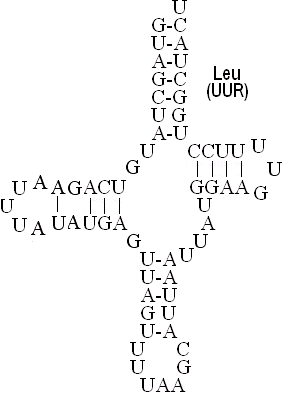

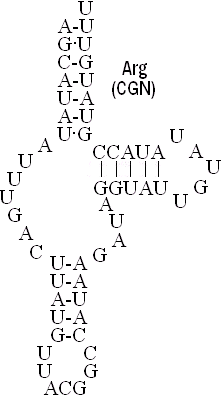

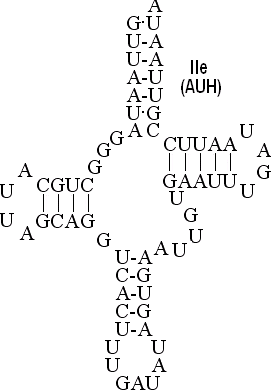

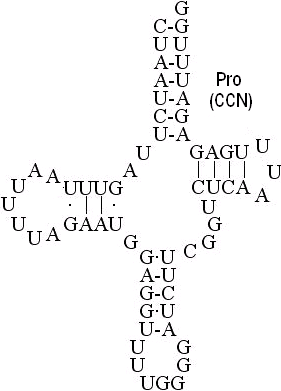

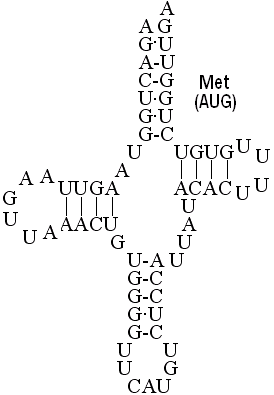

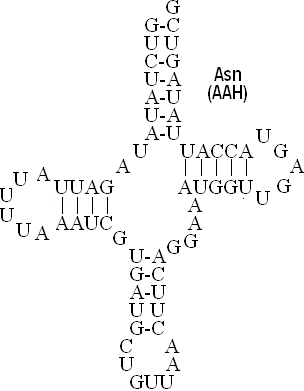

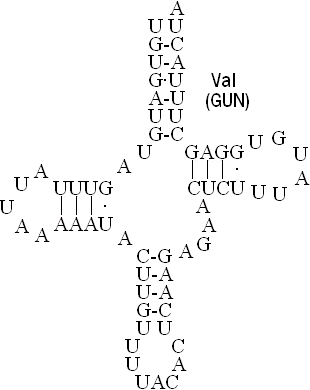

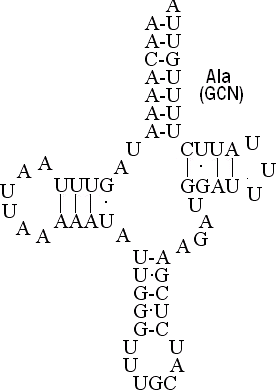

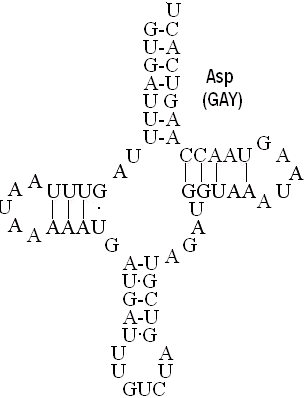

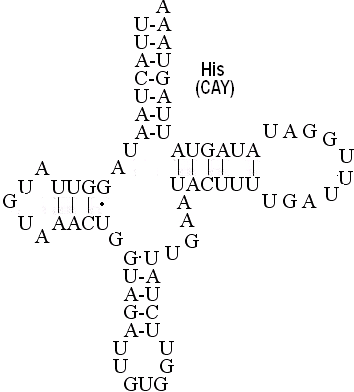

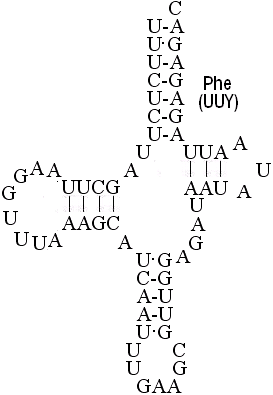

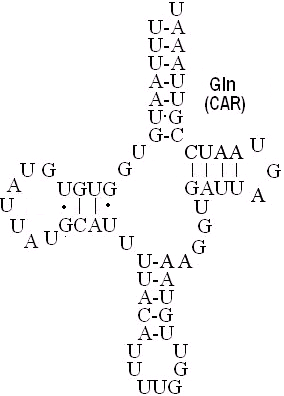

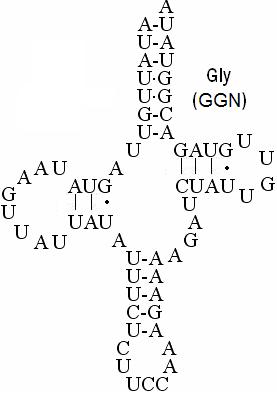


B *T. hydatigena*


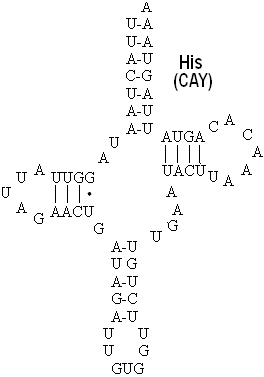

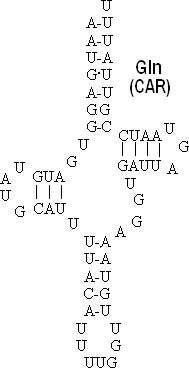

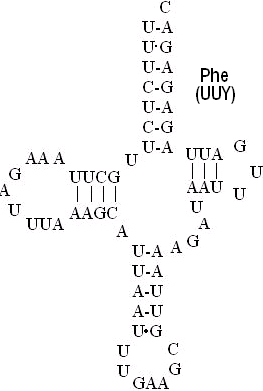

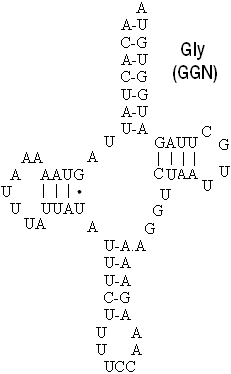

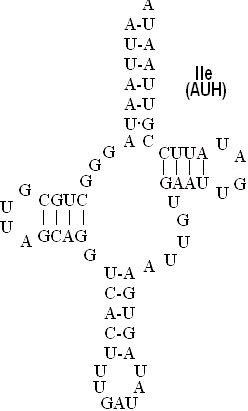

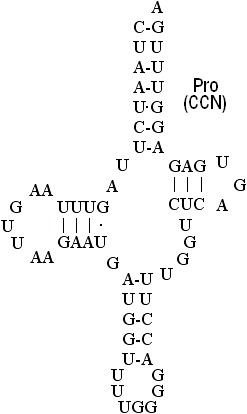

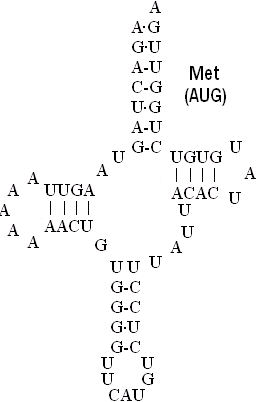

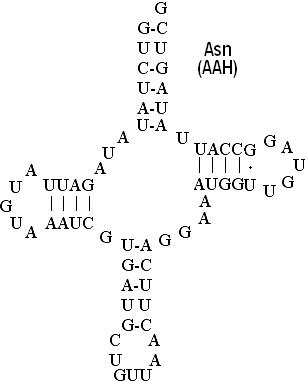

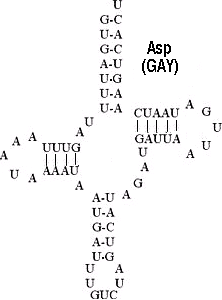

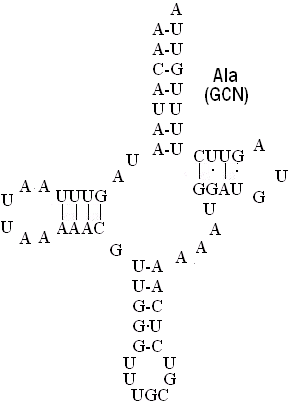

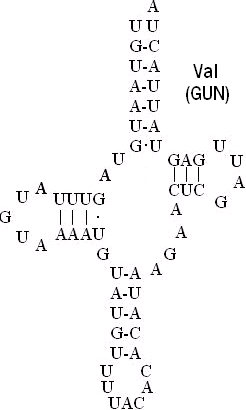

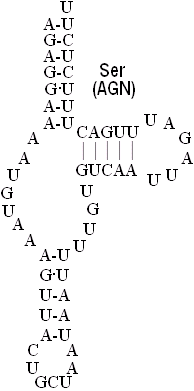

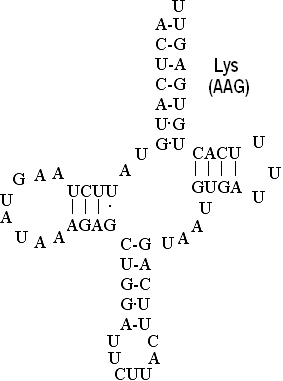

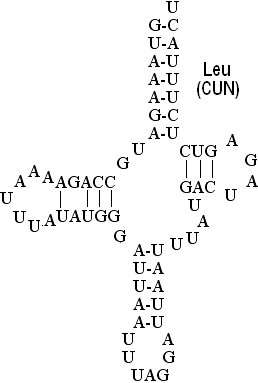

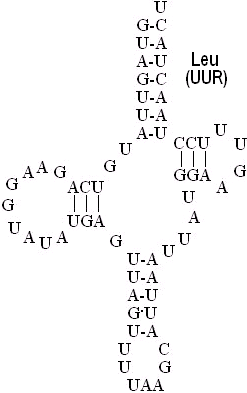

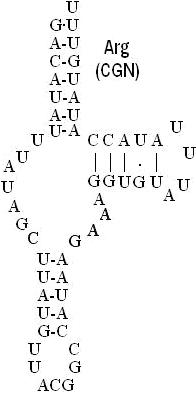

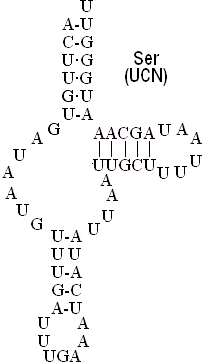

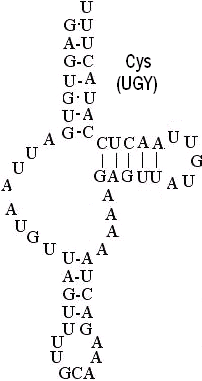

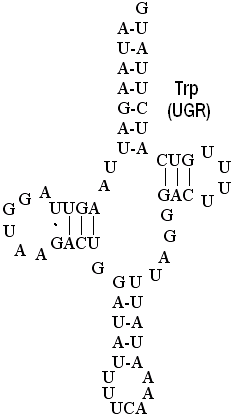

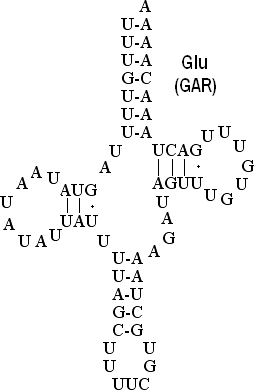

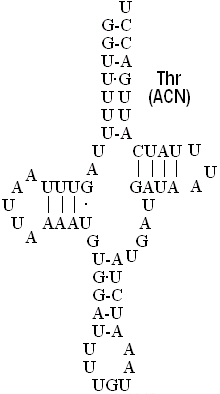


C *T. pisiformis*


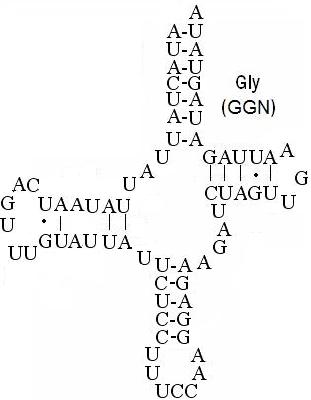

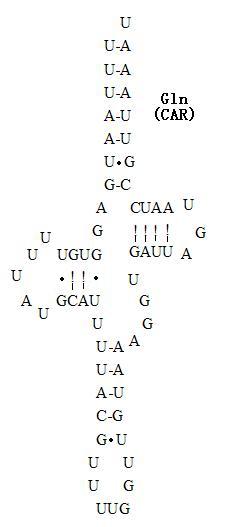

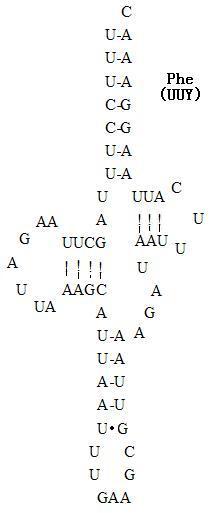

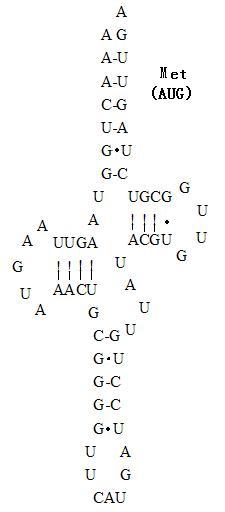

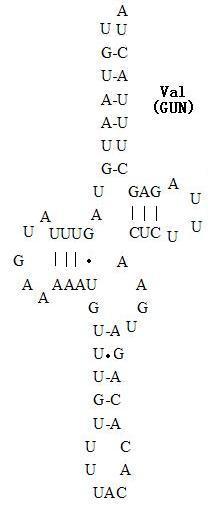

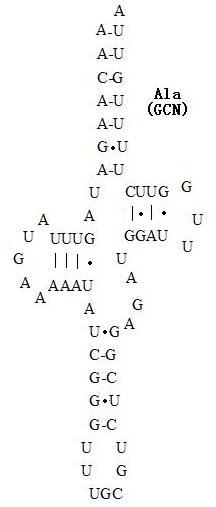

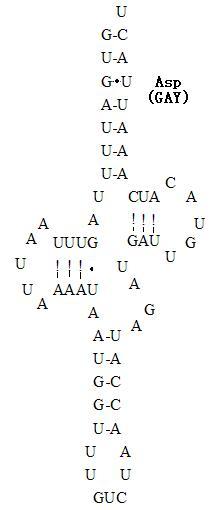

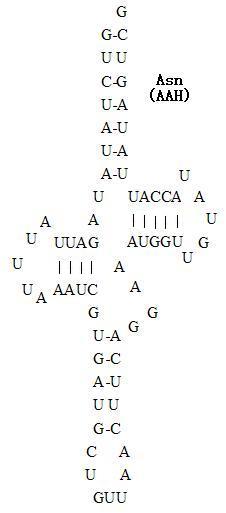

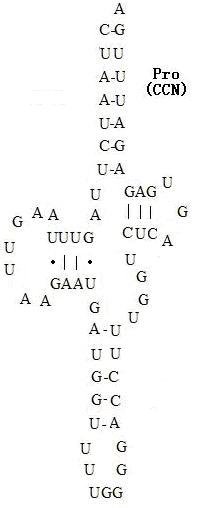

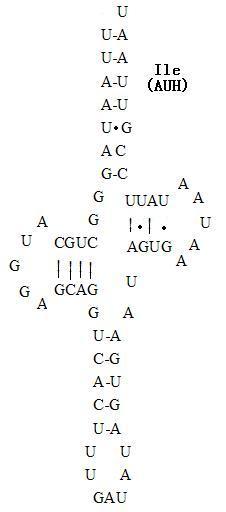

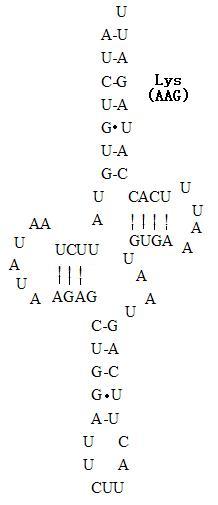

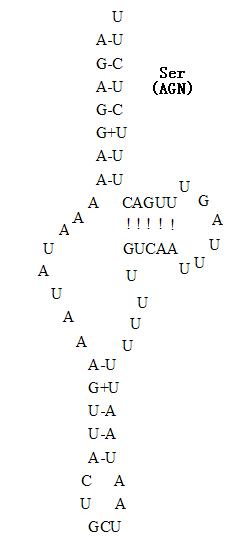

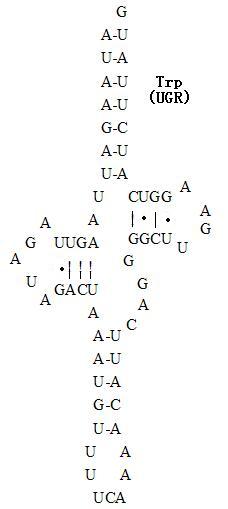

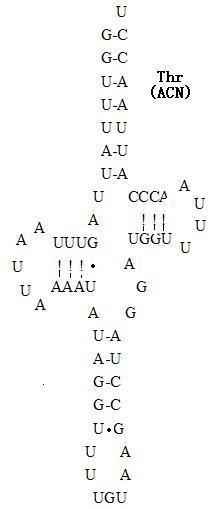

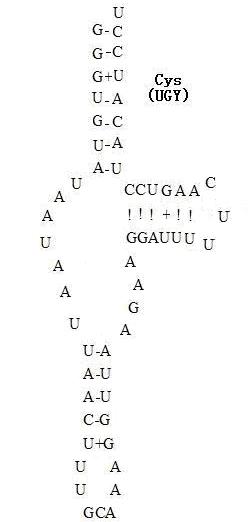

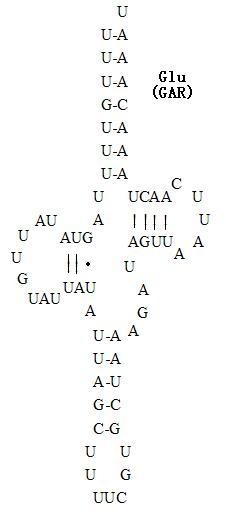

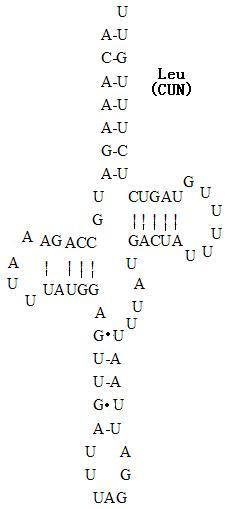

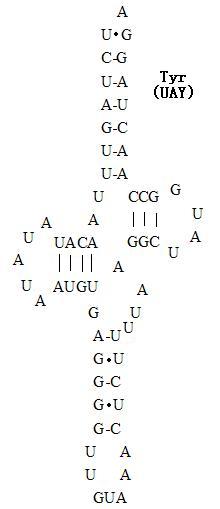

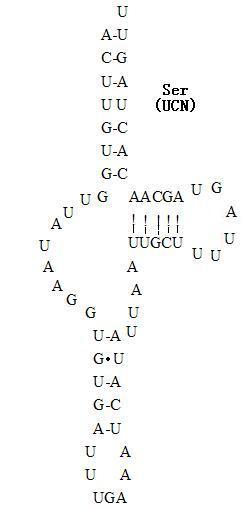

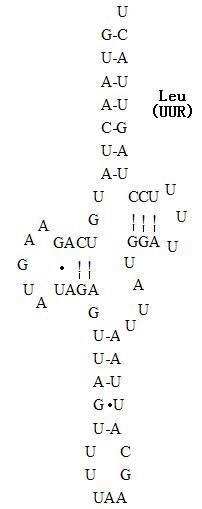

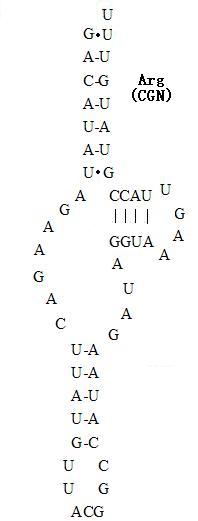

Supplement: Additional file 3 — Predicted secondary structures of tRNAs from A: T. multiceps, B: T. hydatigena and C: T. pisiformis mtDNAs. [file 1471-2164-11-447-S3.DOC]

Additional file 4

A *T. multiceps*

LNR :


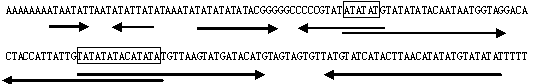


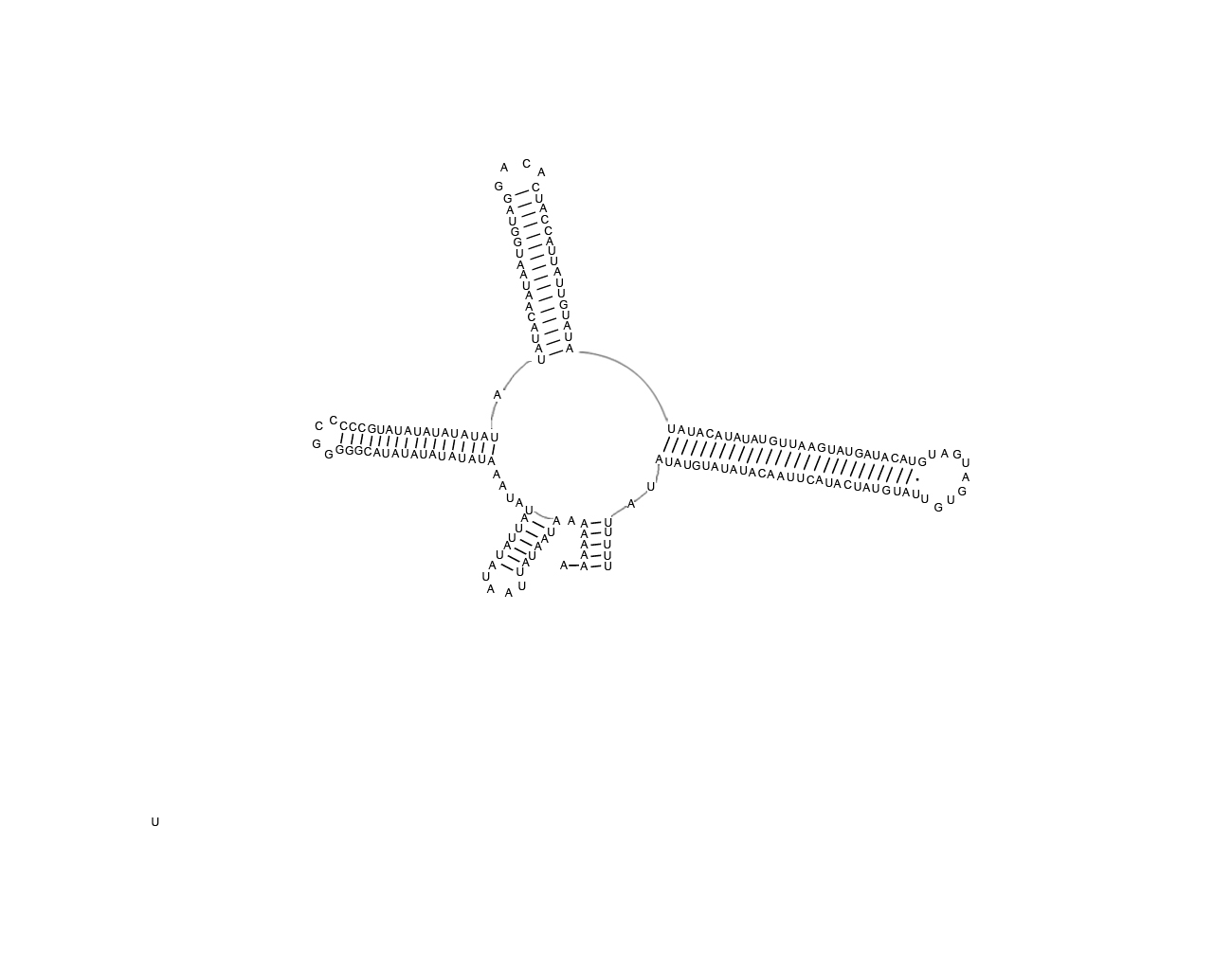


SNR :


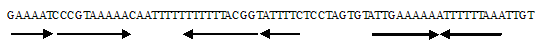


**
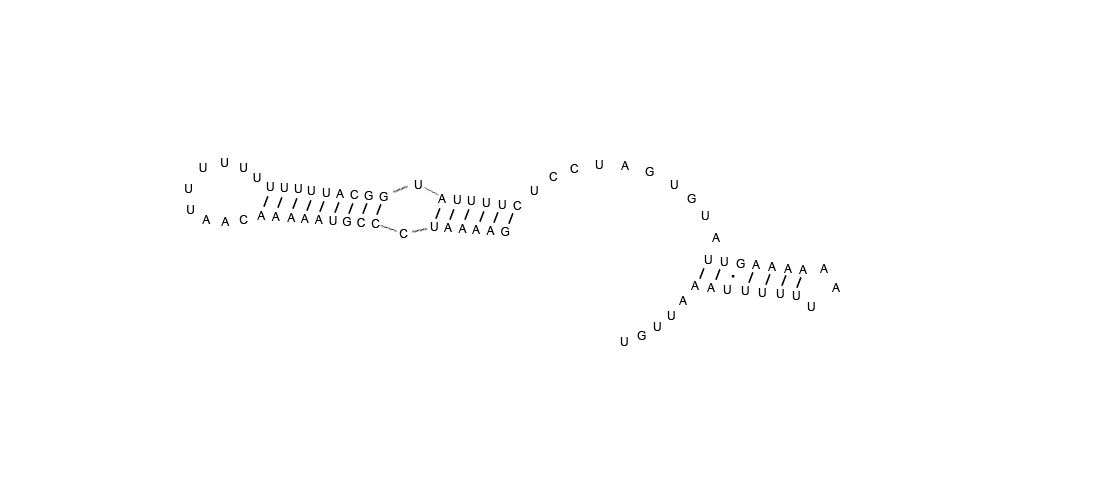
**

B *T. hydatigena*

LNR :


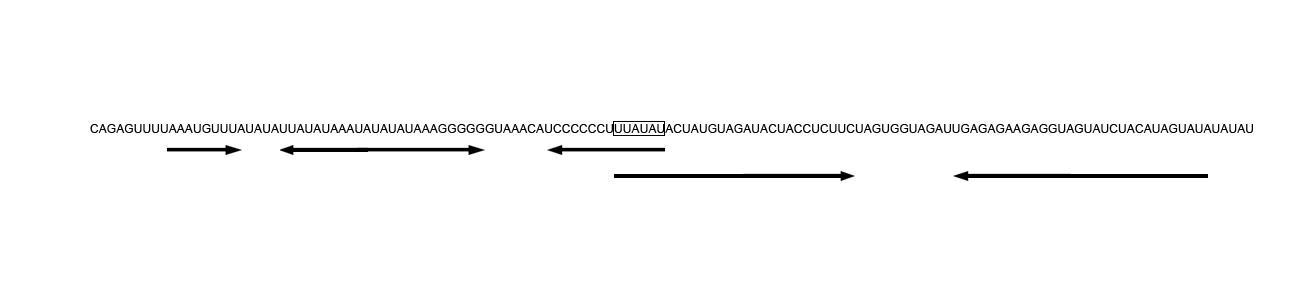


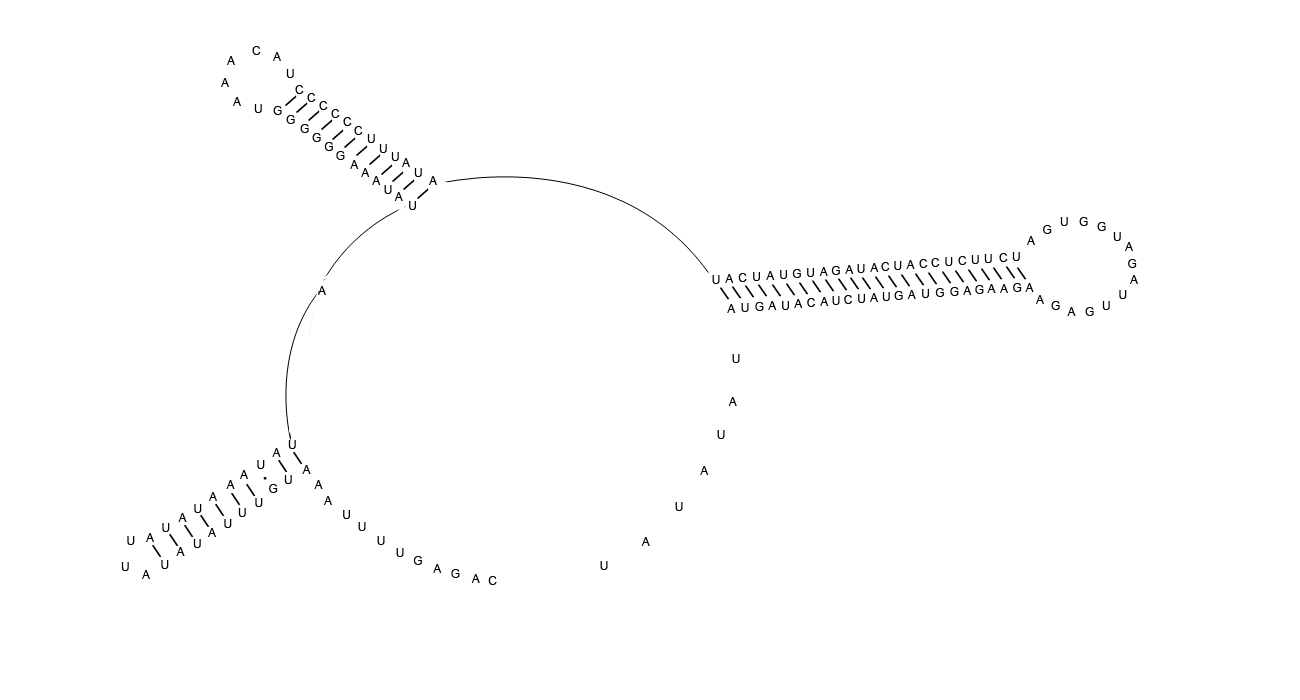


SNR :

**
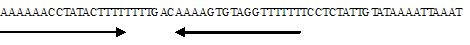
**

**
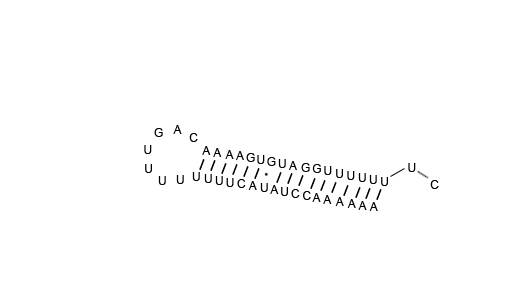
**

C *T. pisiformis*

NR2

**
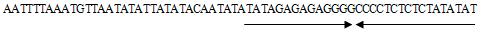
**

**
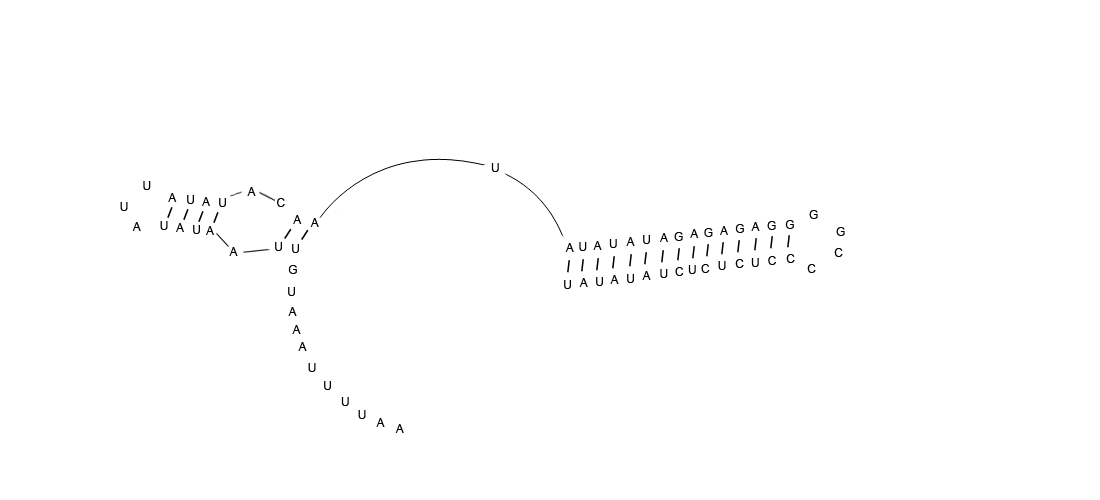
**

NR1

**
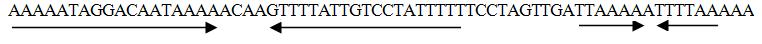
**


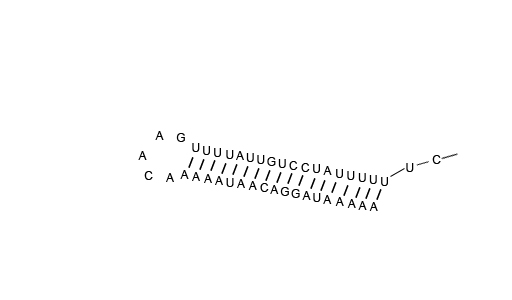

Supplement: Additional file 4 — Sequences and putative structures of LNR and SNR found in the T. multiceps, T. hydatigena and T. pisiformis mtDNAs. Arrows show inverted repeats, which is similar to that of E. multilocularis [50]. Nucleotides in the box are shared by two inverted repeats. A: LNR and SNR sequences of T. multiceps mtDNA and their predicted secondary structures. B: LNR and SNR sequences of T. hydatigena mtDNA and their predicated secondary structures. C: NR1 and NR2 sequences of T. pisiformis mtDNA and their predicated secondary structures. [file 1471-2164-11-447-S4.DOC]
