# Supplementary material for: Complete mitochondrial genomes of Taenia multiceps, T. hydatigena and T. pisiformis: additional molecular markers for a tapeworm genus of human and animal health significance
Source: BMC Genomics. 2010 Jul 22;11:447. doi: 10.1186/1471-2164-11-447 (PMC3091644; doi:10.1186/1471-2164-11-447)
Supplement: Additional file 5 — Primers for amplification of mtDNA fragments and their position in the mt genome of T. pisiformis (Tp). [file 1471-2164-11-447-S5.DOC]

**Additional file 5**

| **Primers** | **Sequences (5’-3’)** | **Position based on**  ***T. pisiformis* mtDNA** |
| --- | --- | --- |
| F1  R1 | CARTTRTTRAGWCAGGGBGTTTCTAAGTG  AAATCCWACWTTYCADACACCCTTCTT | 12576-12604  1117-1091 |
| F2  R2 | TAAACTRRTAGATTGTGGTTCTRTTGAATACT  AAAAYGCYAARCAACGCTTMCCATC | 737-768  2867-2843 |
| F3  R3 | GTGARTCTCCDTATTCTGAGCG  AAAAAACARAARAATAWWACYGGAAACTTCAT | 2433-2454  4459-4428 |
| F4  R4 | AGTTTGGDTTRTTTCCDTTTRTGTT  TGACACRAAATTATTAGCAGTAACTYCACA | 4342-4366  6717-6688 |
| F5  R5 | CGTGATGCTGTTAACTTCARGAAATGG  CCYAAACADACTATWGAAAACATAGC | 6174-6200  7656-7631 |
| F6  R6 | TGATCGTAAATTTAGWTCWGCATTTTTTGATC  TCACGTCAAACCATTCARACAAGCC | 7438-7469  9058-9034 |
| F7  R7 | TTATTTCTCAGGGTCTTTCCGTCTGTTTA  CTTCAAATTCATTTAAAGTTACCTTGTTACG | 8889-8918  10199-10169 |
| F8  R8 | TGACAGTGATTAGATACCCCATTA  TTYCTTTTGGCCGTAACATAAG | 9793-9816  11734-11713 |
| F9  R9 | ATATGAGTTAGTTTTAAGCATTAATTATGG  TGCTTAGTAAAAAAYACTCCWATAAATGG | 11658-11683  12773-12745 |

Note: B=C/G/T; D=A/T/G; M=A/C; N=A/T/G/C; R=A/G; W=A/T; Y=T/C
